# Supplementary material for: Enhancing aortic valve drug delivery with PAR2-targeting magnetic nano-cargoes for calcification alleviation
Source: Nat Commun. 2024 Jan 16;15:557. doi: 10.1038/s41467-024-44726-0 (PMC10792006; doi:10.1038/s41467-024-44726-0)

Supplementary Information

**Enhancing aortic valve drug delivery with PAR2-targeting magnetic nano-cargoes for calcification alleviation**

Supplementary Figures 1–24

Supplementary Tables 1–4

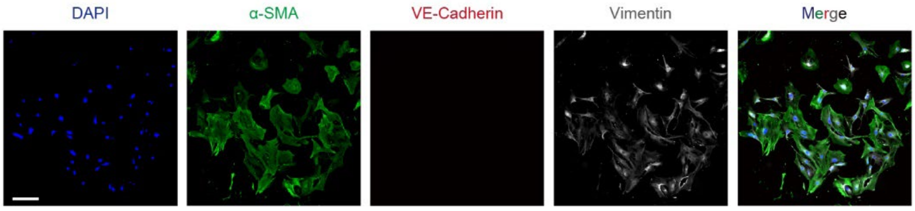


**Supplementary Fig. 1. Characterization of isolated valvular interstitial cells (VICs).** Immunocytofluorescence staining of vimentin, α-SMA, and VE-Cadherin in the VICs isolated from human aortic valves. Scale bar = 200 μm; experiment was repeated independently for 3 times.

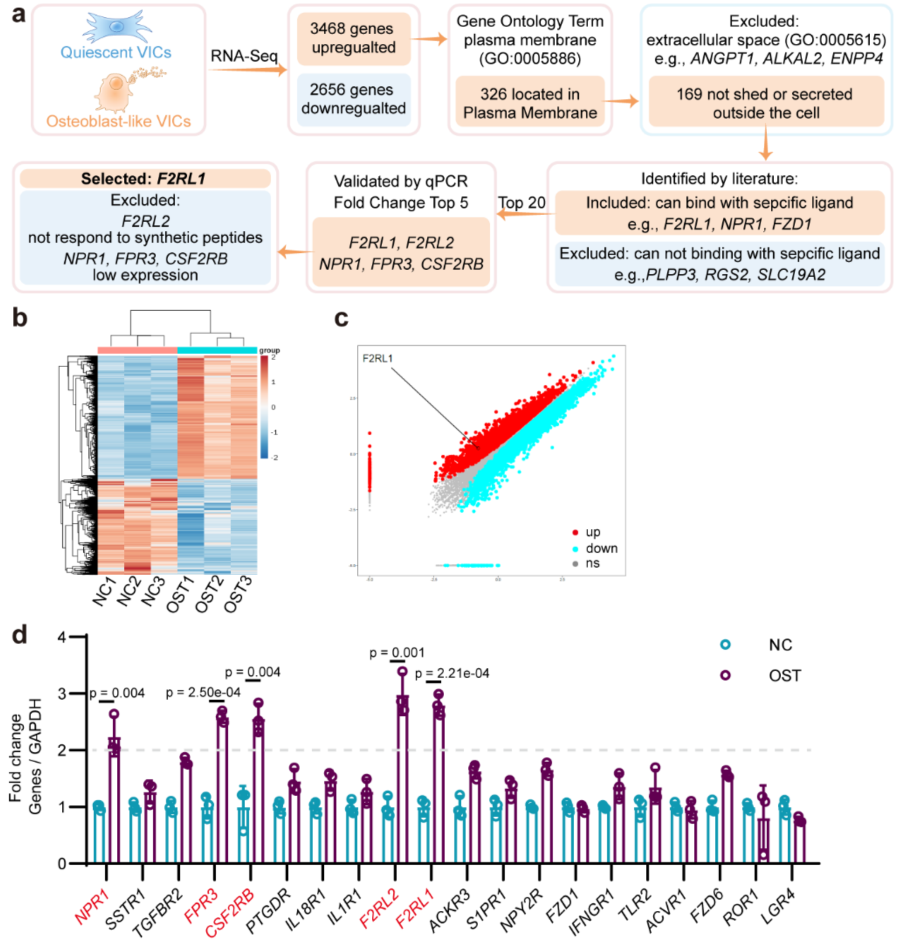


**Supplementary Fig. 2. RNA-seq results of quiescent hVICs and osteogenically differentiated hVICs and validation by quantitative polymerase chain reaction (qPCR).** (a) Flowchart for identifying PAR2 as a target of calcified aortic valve disease (CAVD). (b) flClustered heatmap shows differential gene expression between quiescent hVICs and osteogenically differentiated hVICs. (c) Volcano plot of differential gene expression. Red: up-regulated genes, blue: down-regulated genes, gray: not significant. Adjusted $p$ value < 0.01, log2 Fold Change < -1 or > 1, $n$ = 3 independent samples. (d) QPCR validation of top 20 up-regulated plasma membrane proteins in osteogenically differentiated hVICs compared with quiescent hVICs. Values are presented as mean ± SD.

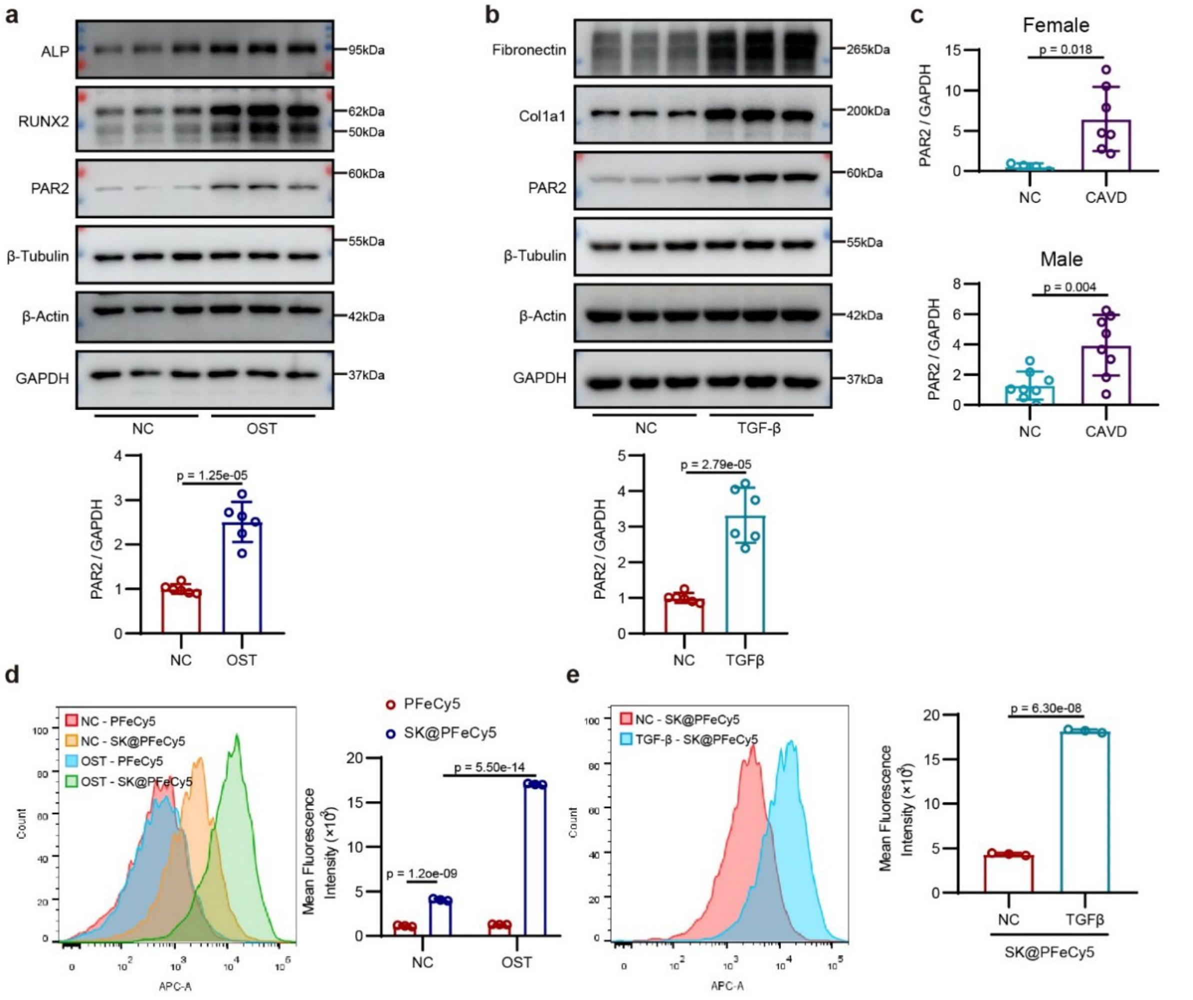


**Supplementary Fig. 3. Targeting ability validation of PAR2-ligand functionalized nanoparticles towards female patients derived hVICs.**

(a) Immunoblot and quantification of PAR2, RUNX2, and ALP in hVICs derived from female patients cultured with osteogenic induction medium for 4 days; $n$ = 6 independent samples. (b) Protein expression of PAR2 in female patient-derived hVICs undergoing myofibrogenic induction for 48 h; $n$ = 6 independent samples. (c) Immunoblot quantification of PAR2 in human aortic valves from female and male patients; female: $n$ = 4 for NC, $n$ = 7 for CAVD; male: $n$ = 8 for NC, $n$ = 8 for CAVD. (d) Flow cytometry histogram and quantification of the PFeCy5 or SK@PFeCy5 uptake by osteogenically differentiated hVICs; $n$ = 3 independent samples. (e) Flow cytometry histogram and quantification of SK@PFeCy5 uptake by

myofibrogenically differentiated hVICs; $n = 3$ independent samples. Values are presented as mean ± SD.

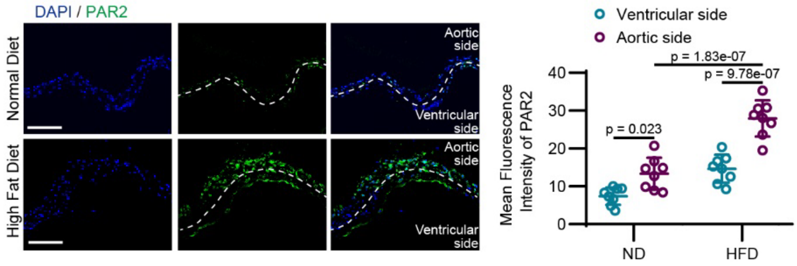


**Supplementary Fig. 4. Expression distribution of PAR2 on the mouse aortic valves.**

PAR2 fluorescence intensity distribution on the aortic valves of ND-fed and HFD- *Ldlr*$^{-/-}$ mice. Scale bar = 100 μm; *n* = 8 independent animals. Values are presented as mean ± SD.

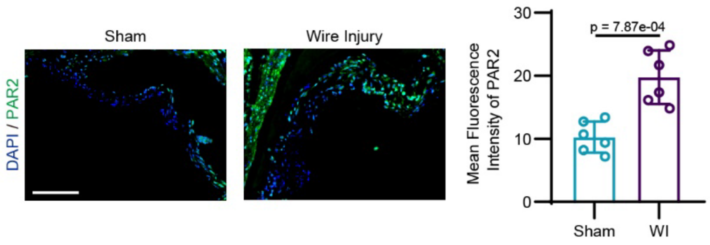


**Supplementary Fig. 5. PAR2 expression in the mouse aortic valve leaflets injured by wire.**

PAR2 expression in the aortic valve of wild-type mice undergoing wire injury or sham. Scale bar = 100 µm; *n* = 6 independent animals. Values are presented as mean ± SD.

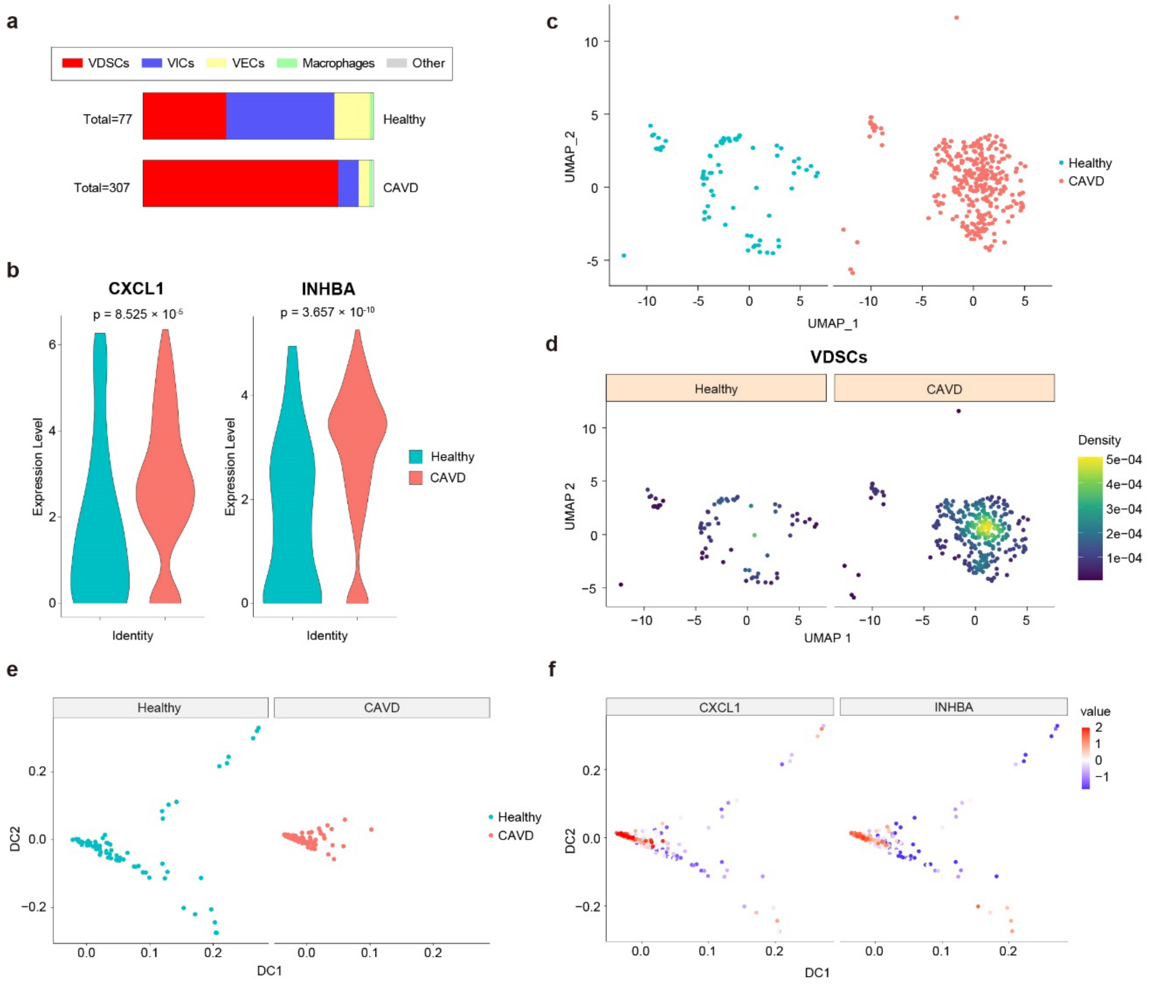


**Supplementary Fig. 6. Transcriptome characterization of *F2RL1*-positive cells within aortic valves via scRNA-seq dataset analysis.**

(a) Cell component of *F2RL1* positive cells in aortic valve (VDSCs, valve-derived stromal cells; VECs, valvular endothelial cells). (b) Violin plot showing *INHBA* and *CXCL1* expression between *F2RL1* positive cells from healthy and calcified valves. (c) UMAP plot of *F2RL1*-positive cells. (d) Scatterplots showing the expression and distribution of VDSC-related genes in the UMAP map. (e) *F2RL1*-positive cell lineage trajectory as reconstructed with destiny. (f) Expression of *CXCL1* and *INHBA* in the *F2RL1*-positive cell lineage trajectory.

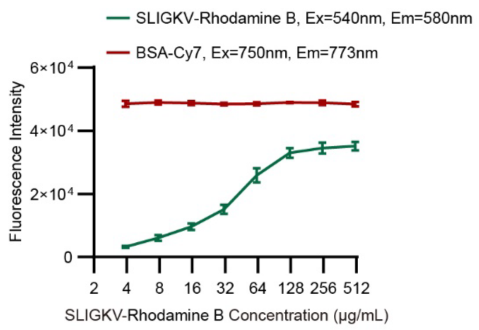


**Supplementary Fig. 7. Efficiency of different SLIGKV-$NH_2$ concentrations in conjugation with nanoparticles**.

Fluorescence intensity of the Cy7 (BSA, indicate nanoparticle concentration) and Rhodamine B (indicate SLIGKV-$NH_2$ concentration) in nanoparticles synthesized with different concentrations of SLIGKV-$NH_2$; $n$ = 3 independent samples. Data are shown as the mean ± SD.

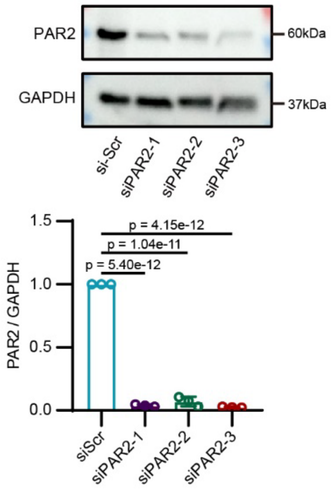


**Supplementary Fig. 8. Identification of siRNA-mediated PAR2 knockdown efficiency.**

PAR2 protein expression in VICs transfected with scramble siRNA or three different siRNA sequences targeting PAR2; *n* = 3 independent samples. Data are shown as the mean ± SD.

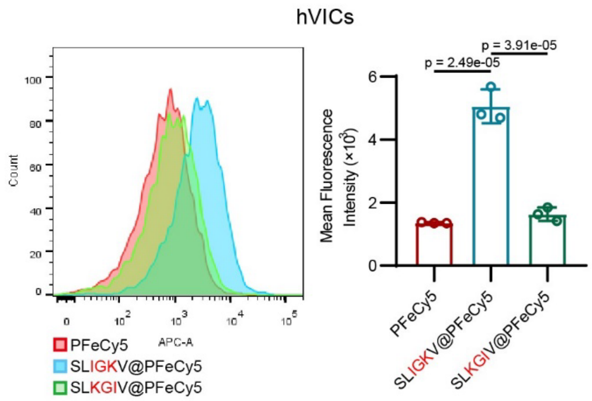


**Supplementary Fig. 9. Targeting ability of mismatched PAR2-ligand functionalized nanoparticles toward hVICs.**

Flow cytometry histogram and quantification of SLIGKV-$NH_2$ or SLKGIV-$NH_2$ functionalized nanoparticle uptake by hVICs; $n$ = 3 independent samples

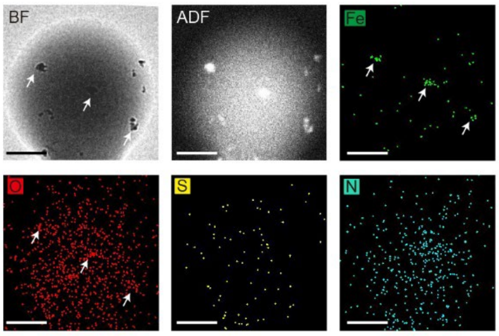


**Supplementary Fig. 10. Element mapping confirming the existence of $Fe_3O_4$ within SK@PFeXCT.**

Typical TEM image, dark-field TEM image, and TEM-EDS of SK@PFeXCT. Scale bar = 50 nm; experiment was repeated independently for 3 times.

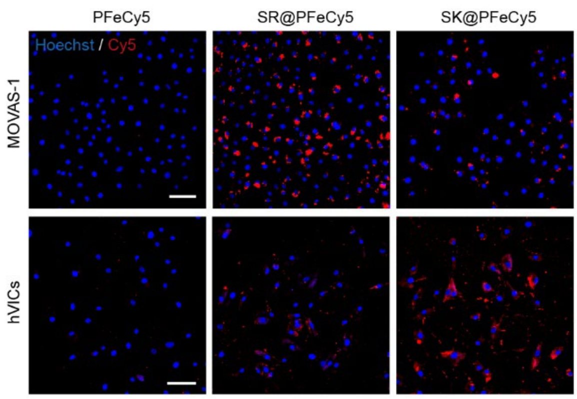


**Supplementary Fig. 11. Species-specific of PAR2-binding ligands in human and mice.**

Fluorescent images of mouse aortic vascular smooth muscle cells (MOVAS-1, upper; scale bar = 10 μm) and hVICs (lower; scale bar = 100 μm) incubated with SR@PFeCy5 or SK@PFeCy5 nanoparticles at 200 μg/mL for 6 h; experiment was repeated independently for 3 times.

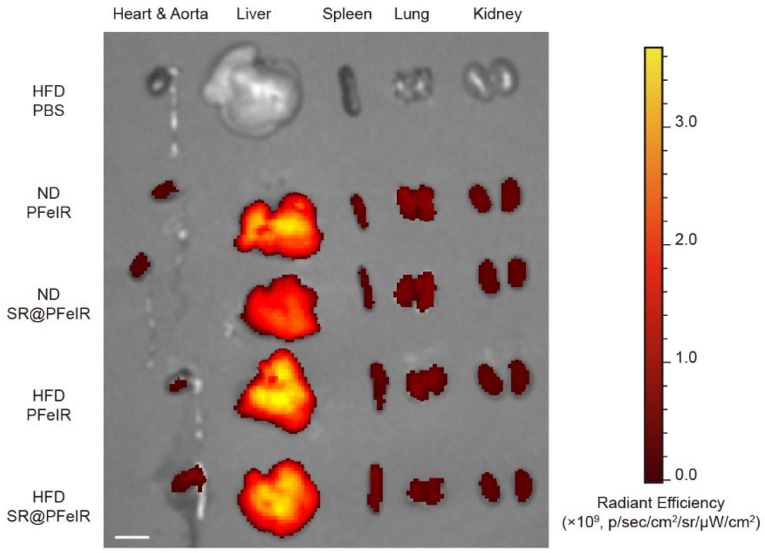


**Supplementary Fig. 12. Biodistribution of SR@PFeIR *in vivo*.**

IVIS images of major organs 4 h post-nanoparticle injection. Scale bar = 1 cm; experiment was repeated independently for 3 times.

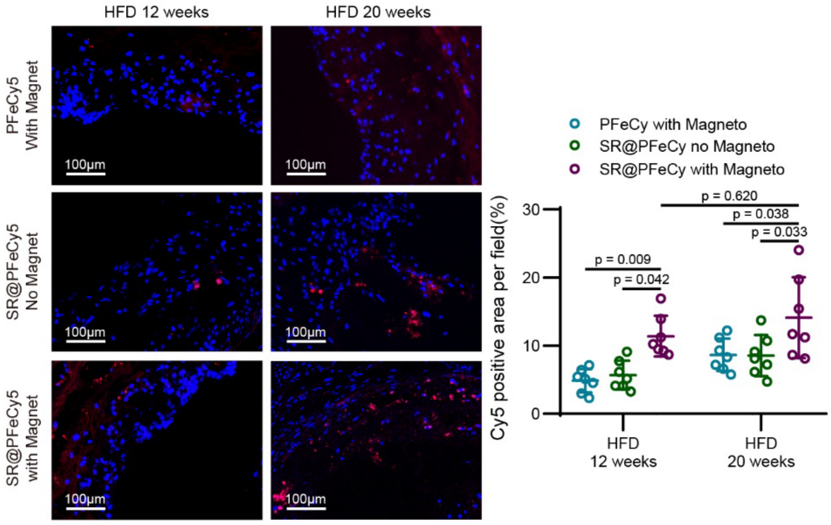


**Supplementary Fig. 13. SR@PFeCy5 is enriched in atherosclerotic aortic sinus.**

Fluorescence images of aortic sinus of HFD fed *Ldlr*$^{-/-}$ mice 4 h post-nanoparticle injection; *n* = 7 independent animals.

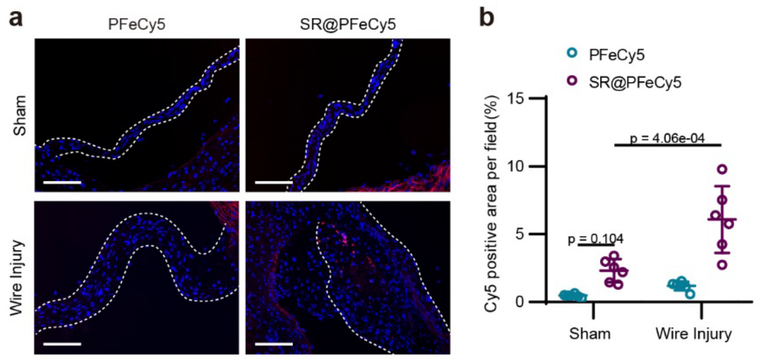


**Supplementary Fig. 14. SR@PFeCy5 targeting the aortic valve injured by wire.**

(a) Fluorescence images of aortic valves in the wire-injury-induced aortic stenosis group or sham group injected with PFeCy5 or SR@PFeCy5. Scale bar = 100 μm. (b) Quantification of the Cy5 positive area in valve cross-section of the normal and injured models; $n = 6$ independent animals. Values are presented as mean ± SD.

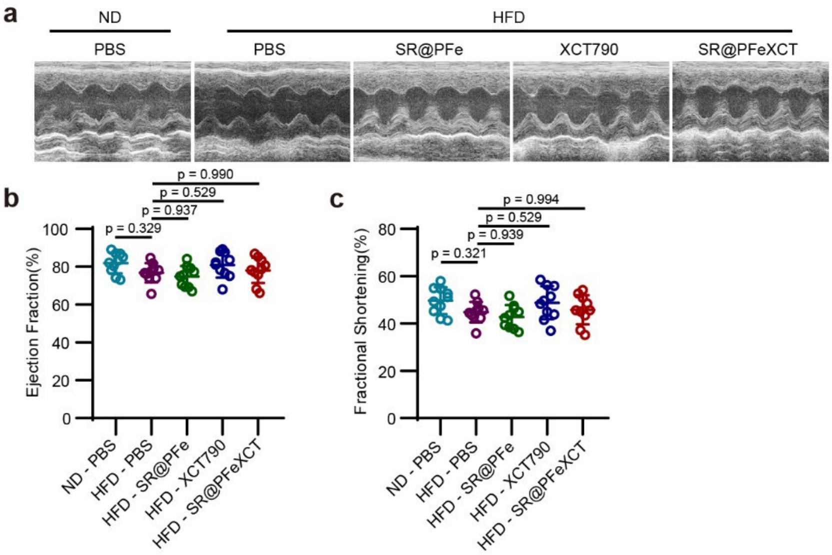


**Supplementary Fig. 15. Nanoparticle application does not affect cardiac function in HFD-fed *Ldlr*$^{-/-}$ mice.**

(a) Typical M-mode echocardiographic images of *Ldlr*$^{-/-}$ mice. (b) Ejection fraction (EF) and (c) fractional shortening (FS) of *Ldlr*$^{-/-}$ mice; *n* = 10 independent animals. Values are presented as mean ± SD.

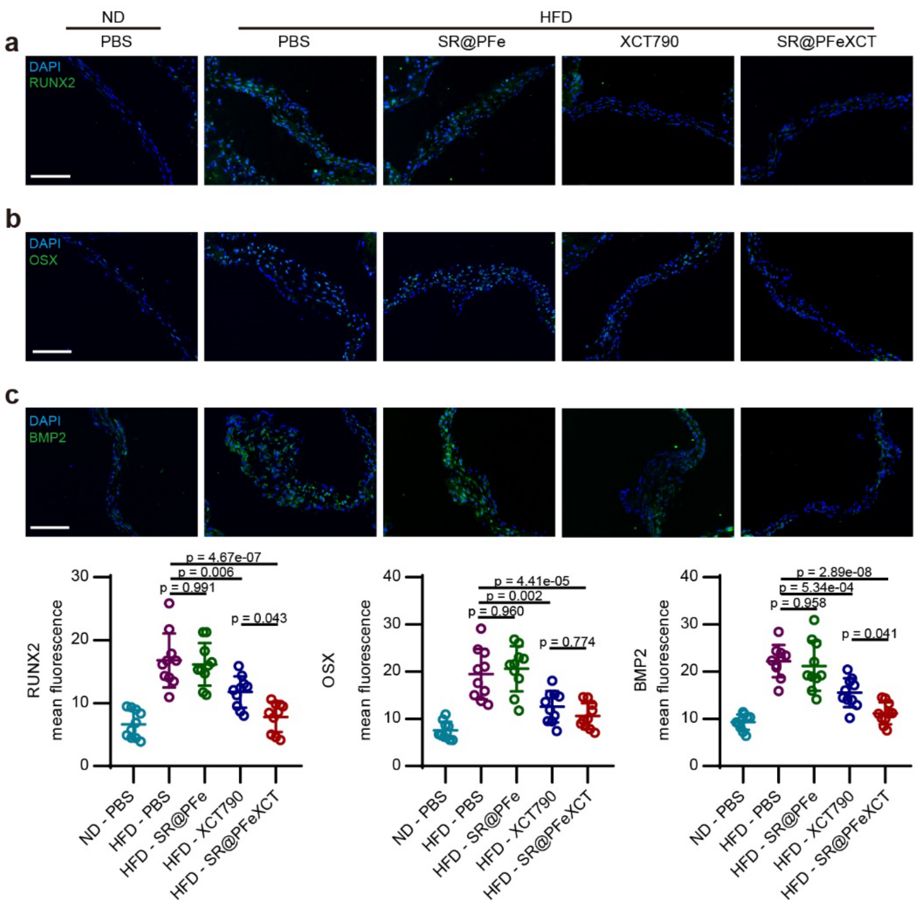


**Supplementary Fig. 16. SR@PFeXCT alleviates the osteogenic differentiation of VICs in *Ldlr*$^{-/-}$ mice.**

Immunofluorescence staining and quantification of (a) RUNX2, (b) OSX, and (c) BMP2 in aortic valves of *Ldlr*$^{-/-}$ mice from ND group, HFD group, HFD plus XCT790 injection group, HFD plus SR@PFe nanoparticle injection group, and HFD plus SR@PFeXCT nanoparticle injection group. Scale bar = 100 μm; *n* = 10 independent animals. Data are shown as means ± SD.

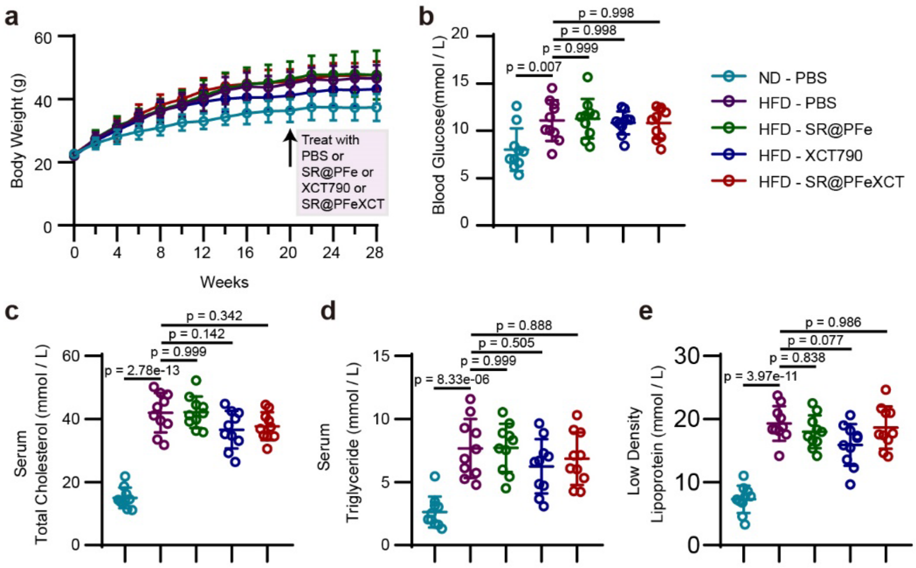


**Supplementary Fig. 17. Metabolic parameters in different groups of mice.**

(a) Body mass of Ldlr$^{-/-}$ mice in different groups. (b) Blood glucose, (c) serum total cholesterol level, (d) serum total triglycerides, and (e) serum low-density lipoprotein cholesterol level of Ldlr$^{-/-}$ mice after 28 weeks of HFD feeding. Data are shown as means ± SD.

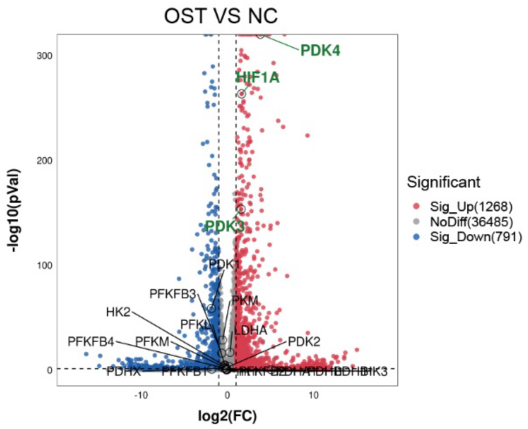


**Supplementary Fig. 18. *HIF1A*, *PDK3,* and *PDK4* are upregulated in osteogenically differentiated hVICs.**

Volcano plot showing differential gene expression between quiescent hVICs and osteogenically differentiated hVICs; glycolysis-related genes are labeled. Red: up-regulated genes, blue: down-regulated genes, gray: not significant. Adjusted p value < 0.01, log2 Fold Change < -1 or > 1; $n = 4$ independent samples.

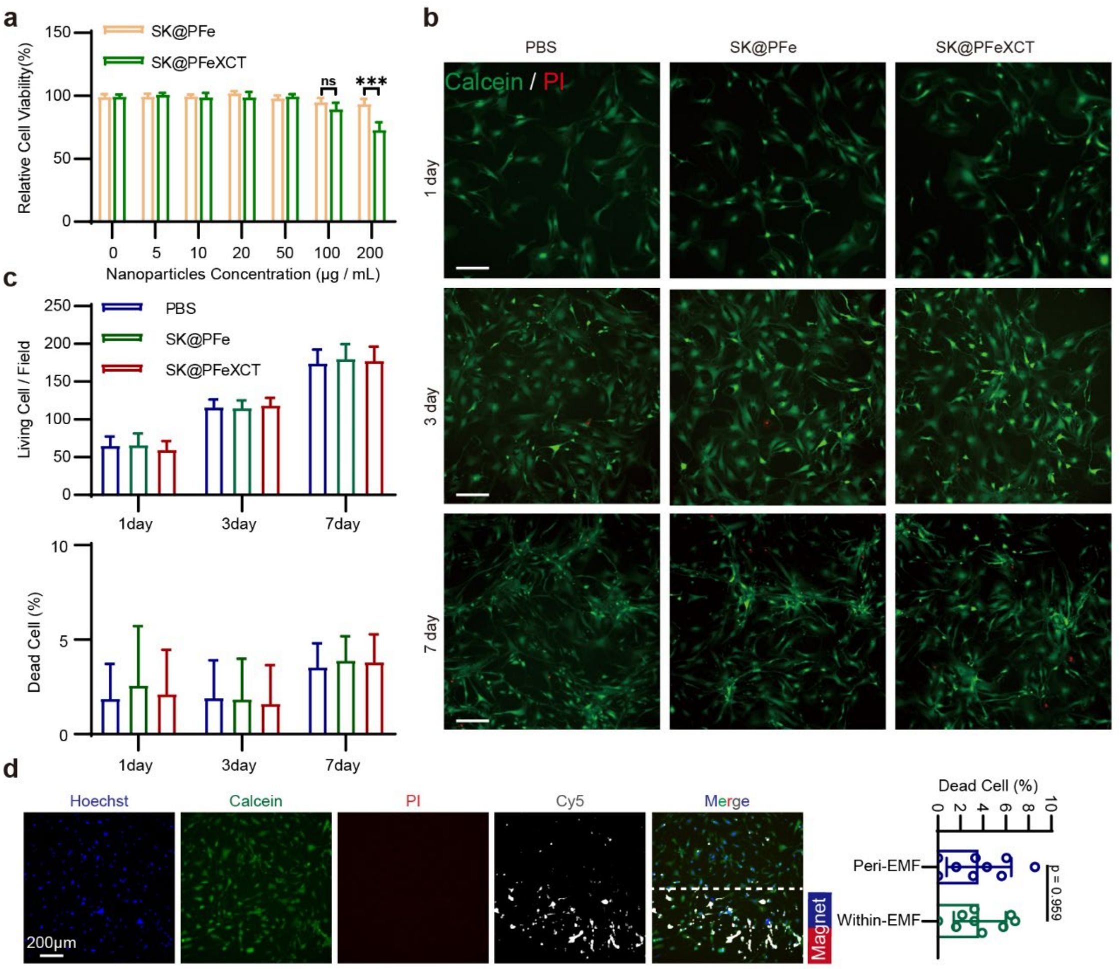


**Supplementary Fig. 19. *In vitro* biocompatibility of SK@PFeXCT.**

(a) Representative CCK-8 results of hVICs treated with SK@PFe NPs or SK@PFeXCT NPs at different concentrations for 6 h. (b) Representative calcein-AM/PI staining of hVICs at day 1, day 3, and day 7 after incubation with SK@PFe nanoparticles or SK@PFeXCT nanoparticles at 20 μg/mL. Viable cells stained with Calcein-AM appear in green, and dead cells stained with PI in red. Scale bar = 200 μm. (c) Quantitative results of live/dead staining. Ten visual fields were randomly chosen for each group. (d) Representative calcein-AM/PI staining of hVICs incubation with SK@PFe nanoparticles under EMF. Scale bar = 200 μm. Data are shown as the mean ± SD.

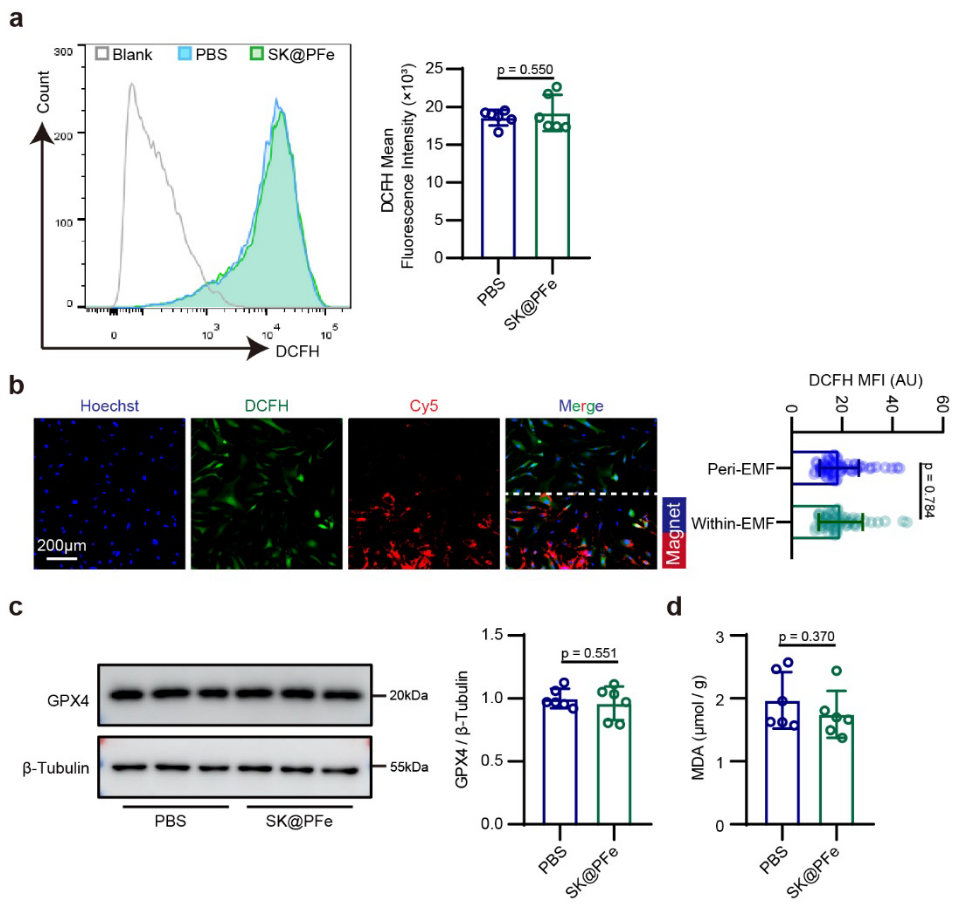


**Supplementary Fig. 20. ROS production and ferroptosis of hVICs incubated with SK@PFe nanoparticles.**

(a) Flow cytometry histogram and quantification of DCFH fluorescence intensity of hVICs incubated with or without SK@PFe nanoparticles; *n* = 6 independent samples. (b) Representative DCFH staining of hVICs incubated with SK@PFe nanoparticles under the EMF. Scale bar = 200 μm. (c) Western blot and quantification of GPX4 in hVICs cultured with or without SK@PFe nanoparticles; *n* = 6 independent samples. (d) Malondialdehyde (MDA) content in hVICs cultured with or without SK@PFe nanoparticles; *n* = 6 independent samples.

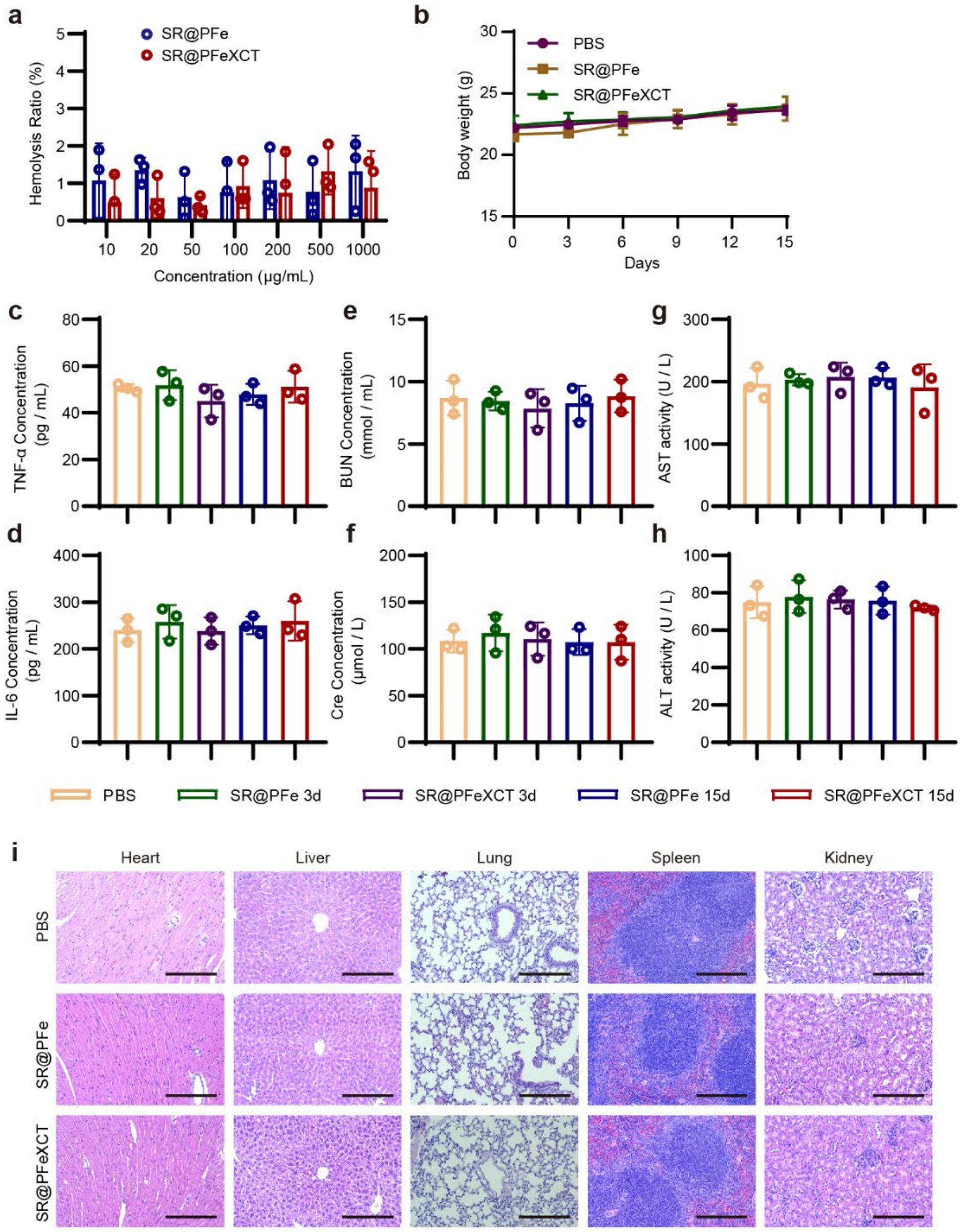


**Supplementary Fig. 21.** ***In vivo*** **biocompatibility of SR@PFeXCT.**

(a) Hemolysis results of SR@PFe or SR@PFeXCT nanoparticles at different concentrations. Phosphate buffer saline (PBS) served as a negative control, and ultra-pure water as a positive control. (b) Body weights of mice injected with PBS, SR@PFe, or SR@PFeXCT nanoparticles. Injection was performed on day 0 and day 7 at 15 mg/kg. Serum levels of inflammatory factors [(c) tumor necrosis factor-alpha and (d) interleukin-6], kidney function indicators [(e) blood urea nitrogen and (f) creatinine], and liver function indicators [(g)

aspartate transaminase and (h) alanine transaminase] of mice injected with PBS, SR@PFe or SR@PFeXCT nanoparticles. Injection was performed on day 0 and day 7 at 15 mg/kg. (i) Representative hematoxylin and eosin (H&E) staining of major organs of mice injected with PBS, SR@PFe or SR@PFeXCT nanoparticles; experiment was repeated independently for 3 times. Injection was performed on day 0 and day 7 at 15 mg/kg, and mice were euthanized 15 days after the first injection. Scale bar = 200 μm, $n$ = 3 independent animals. Data are shown as means ± SD.

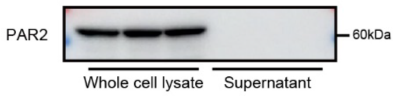


**Supplementary Fig. 22. Intracellular and extracellular protein expression of PAR2.**

Immunoblot of PAR2 in whole cell lysate and supernatant of cultured hVICs.

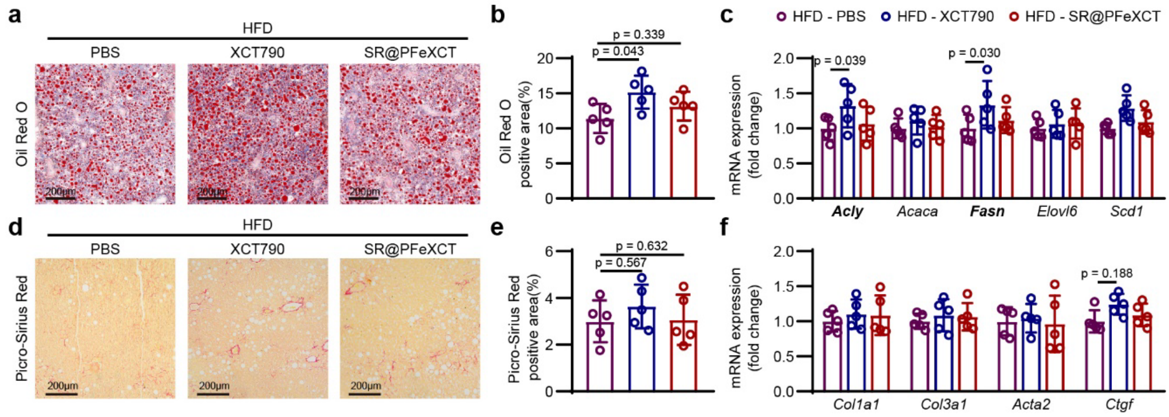


**Supplementary Fig. 23. Liver steatosis and fibrosis in HFD-fed *Ldlr*$^{-/-}$ mice**

(a) Representative Oil Red O staining and (b) quantification of liver sections obtained from HFD-fed *Ldlr*$^{-/-}$ mice; scale bar = 200 μm; *n* = 5 independent animals. (c) Adipogenic mRNA expression in the liver of HFD-fed *Ldlr*$^{-/-}$ mice; *n* = 5 independent animals. (d) Representative Picro-Sirius Red staining and (e) quantification of liver sections; scale bar = 200 μm; *n* = 5 independent animals. (e) Profibrotic genes expression in the liver of HFD-fed *Ldlr*$^{-/-}$ mice; *n* = 5 independent animals.

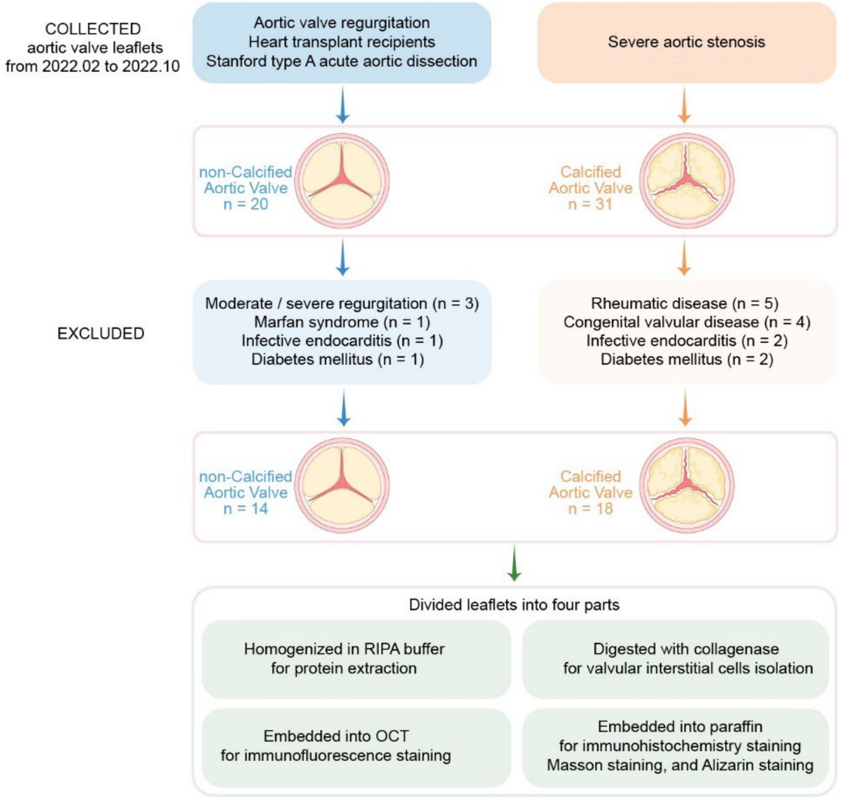


**Supplementary Fig. 24. Flowchart of the patients' tissues included and excluded in the study.**

Calcified aortic valve leaflets were obtained from patients with severe aortic stenosis who underwent aortic valve replacement surgery, while non-calcified aortic valves were obtained from heart transplant recipients, patients with Stanford type A acute aortic dissection or aortic valve regurgitation. Valves from patients with severe aortic regurgitation, rheumatic disease, infective endocarditis, congenital valvular disease, or diabetes were excluded. Leaflets were divided into four pieces: (1) digested with collagenase type II for VICs isolation and culture. (2) homogenized in radioimmunoprecipitation assay (RIPA) buffer for protein extraction and immunoblot; (3) embedded in paraffin for immunohistochemistry staining; (4) embedded in optimal cutting temperature compound for immunofluorescence staining.

**Supplementary Table1** Baseline clinical and echocardiographic parameters of the donors of tissues used for immunoblot, immunohistochemistry, immunofluorescence and cell isolation

| parameters | Control□ n=14□ | CAVD□ n=18□ | p Value |
|---|---|---|---|
| Age | 59.93 ± 16.07 | 61.44 ± 7.310 | 0.7238 |
| Sex, (Female, %) | 4□ 28.57%□ | 7□ 38.89%□ | N/A |
| BMI (kg/m$^2$) | 22.99 ± 3.787 | 22.27 ± 2.679 | 0.5357 |
| Diabetes, n (%) | 0 | 0 | N/A |
| Hypertension, n (%) | 7□ 50%□ | 6□ 33.33%□ | N/A |
| Smoking, n (%) | 5□ 35.71%□ | 4□ 22.22%□ | N/A |
| Coronary heart disease, n (%) | 2□ 14.29%□ | 4□ 22.22%□ | N/A |
| Bicuspid aortic valves, n (%) | 0 | 0 | N/A |
| LVEF□ %□ | 55.51 ± 9.498 | 61.37 ± 8.745 | 0.0860 |
| Aortic valve area (cm$^2$) | 2.826 ± 0.6929 | 0.8379 ± 0.5897 | 1.17e-05 |
| Aortic valve peak velocity (m/s) | 1.927 ± 0.5202 | 4.435 ± 0.6985 | 2.96e-12 |
| Peak transvalvular pressure gradient (mmHg) | 15.79 ± 8.911 | 80.56 ± 24.52 | 1.96e-10 |
| Mean transvalvular pressure gradient (mmHg) | 7.846 ± 4.506 | 45.89 ± 16.05 | 3.97e-09 |
| LDL (mmol/L) | 2.200 ± 0.5713 | 2.213 ± 0.8200 | 0.9608 |
| HDL (mmol/L) | 1.281 ± 0.3336 | 1.250 ± 0.3166 | 0.7921 |
| Triglycerides (mmol/L) | 1.079 ± 0.3184 | 1.872 ± 2.259 | 0.2035 |
| Total cholesterol (mmol/L) | 4.135 ± 0.7899 | 4.331 ± 0.9416 | 0.5372 |
| Statins, n (%) | 1□ 13.3%□ | 3□ 20%□ | N/A |
| ACEi/ARB, n (%) | 2□ 20%□ | 2□ 10%□ | N/A |

Values are mean ± standard deviation (SD) or %. CAVD, calcified aortic valve disease; BMI, body mass index; LVEF, left ventricular ejection fraction; LDL, low-density lipoprotein cholesterol; HDL, high-density lipoprotein cholesterol; ACEi, angiotensin-converting enzyme inhibitor; ARB, angiotensin receptor blocker.

**Supplementary Table 2** PAR2 expression analysis in immunoblot after Propensity-score matching

| Parameters | Control (n = 6) | CAVD (n = 6) | p Value |
|---|---|---|---|
| Age | 59.5±10.5972 | 62.8333±7.4677 | 0.544 |
| BMI (kg/m$^2$) | 21.655±3.4219 | 22.9983±3.2718 | 0.503 |
| Sex, (Female, %) | 0 (0) | 3 (50) | 0.076 |
| Smoking, n (%) | 1 (16.67) | 3 (50) | 0.262 |
| Bicuspid aortic valves, n (%) | 0 | 0 | N/A |
| Diabetes, n (%) | 0 | 0 | N/A |
| Hypertension, n (%) | 3(50) | 1(16.67) | 0.262 |
| Coronary heart disease, n (%) | 1(16.67) | 1(16.67) | 1.000 |
| LDL (mmol/L) | 2.2033±0.4859 | 2.2083±0.8343 | 0.990 |
| HDL (mmol/L) | 1.4533±0.3154 | 1.35±0.2188 | 0.526 |
| Triglycerides (mmol/L) | 1.1217±0.3773 | 1.1767±0.3018 | 0.786 |
| Total cholesterol (mmol/L) | 4.3883±0.8134 | 4.2817±0.9082 | 0.835 |
| Statin, n (%) | 1(16.67) | 1(16.67) | 1.000 |
| ACEi/ARB, n (%) | 1(16.67) | 1(16.67) | 1.000 |
| **PAR2 expression** | **1.2667±0.9082** | **5.8833±3.0995** | **0.006** |

**Supplementary Table 3** Sequence for siRNA

| siRNA name | | Sequence (5`->3`) |
|---|---|---|
| si-Scramble | Sense | UUCUCCGAACGUGUCACGUTT |
| | Antisense | ACGUGACACGUUCGGAGAATT |
| si-hPAR2-1 | Sense | GGUAAGGUUGAUGGCACAUTT |
| | Antisense | AUGUGCCAUCAACCUUACCTT |
| si-hPAR2-2 | Sense | GGUCACCAUUCCUUUGUAUTT |
| | Antisense | AUACAAAGGAAUGGUGACCTT |
| si-hPAR2-3 | Sense | GCAAAGAACGCUCUCCUUUTT |
| | Antisense | AAAGGAGAGCGUUCUUUGCTT |

**Supplementary Table 4** Primer Sequence for qRT-PCR

| Gene | | Sequence (5`->3`) |
|---|---|---|
| *hGAPDH* | Sense | GGAGCGAGATCCCTCCAAAAT |
| | Antisense | GGCTGTTGTCATACTTCTCATGG |
| *hF2RL1* | Sense | TGGCCATGTACCTGATCTGC |
| | Antisense | TCTGCTTTACAGTGCGGACA |
| *hF2RL2* | Sense | AGGCGAGTCTCCTCATCCTT |
| | Antisense | AGACTACCCAGGCACAAAGC |
| *hNPR1* | Sense | GGAGATTGCCCTGAGGAGTG |
| | Antisense | ATTGTTCGCGTACTGCTCCA |
| *hFPR3* | Sense | GAAACTGAGGAGGTGCTCCC |
| | Antisense | CACCCAGATCACAAGCCCAT |
| *hCSF2RB* | Sense | GCCTTCACTAGCGGGAGTC |
| | Antisense | GATCTGAGGCAGCTGGAGTC |
| *hTGFBR2* | Sense | GCACGTTCAGAAGTCGGATG |
| | Antisense | CTGCACCGTTGTTGTCAGTG |
| *hSSTR1* | Sense | CAGCGCCATCCTGATCTCTT |
| | Antisense | TGTAGATGTTGGTGGCCGTC |
| *hIL18R1* | Sense | CCTTGACCCTTTGGGTGCTT |
| | Antisense | GCAAGTGAACACGAGCAATGT |
| *hIL1R1* | Sense | GGAGACGGAGGACTTGTGTG |
| | Antisense | ATCACAGGCCTTGTGGGTTT |
| *hPTGDR* | Sense | ATGCGCAACCTCTATGCGAT |
| | Antisense | GCGCGATAAATTACGGGCAG |
| *hACKR3* | Sense | CAGAGCCAGGGAACTTCTCG |
| | Antisense | GACGCTTTTGTTGGGCATGT |
| *hFZD1* | Sense | CCTGTTTATCGGCACGTCCT |
| | Antisense | TAGCGTAGCTCTTGCAGCTC |
| *hIFNGR1* | Sense | CAGAGCCAGGGAACTTCTCG |

| | | |
|---|---|---|
| | Antisense | GACGCTTTTGTTGGGCATGT |
| *hS1PR1* | Sense | ATATCAGCGCGGACAAGGAG |
| | Antisense | CTGCCAACAGGTCTGAGAGG |
| *hNPY2R* | Sense | GGCAACTCCTTGGTGATCCA |
| | Antisense | CGGTCCAGGGCAATTACTGT |
| *hTLR2* | Sense | ATGTCACAGGACAGCACTGG |
| | Antisense | CCAGGAATGAAGTCCCGCTT |
| *hACVR1* | Sense | AATCCCCGAGACGTGGAGTA |
| | Antisense | GGAAGACCAGAGCCACTTCC |
| *hFZD6* | Sense | ATGTGGTTCCACCTTGTCGT |
| | Antisense | CATTCAAGCTCCTCAGGCCA |
| *hROR1* | Sense | GTGCCTACCTCATCATGGAACA |
| | Antisense | GAGCCATAGATGGTGGACCG |
| *hLGR4* | Sense | CCCAAGCGCTGGATATCAGTA |
| | Antisense | CGCAAAGACTGCAAAGCACT |
| *hPDK4* | Sense | GGAGCATTTCTCGCGCTACA |
| | Antisense | ACAGGCAATTCTTGTCGCAAA |
| *mGapdh* | Sense | TTTGGCATTGTGGAAGGGCT |
| | Antisense | GTCAGATCCACGACGGACAC |
| *mAcly* | Sense | CCAGCTATGCCCCAAGGAAA |
| | Antisense | GATCAGCACGTCTACCTCCG |
| *mAcaca* | Sense | GCCTCCGTCAGCTCAGATAC |
| | Antisense | GACCACCGACGGATAGATCG |
| *mFasn* | Sense | GGCCCCTCTGTTAATTGGCT |
| | Antisense | GGATCTCAGGGTTGGGGTTG |
| *mElovl6* | Sense | CTGGATGCAGCATGACAACG |
| | Antisense | GCCGATGTAGGCCTCAAAGA |
| *mScd1* | Sense | CCCTCCGGAAATGAACGAGA |
| | Antisense | CAGAGCGCTGGTCATGTAGT |

| | | |
|---|---|---|
| *mCol1a1* | Sense | TTCTCCTGGCAAAGACGGAC |
| | Antisense | CTCAAGGTCACGGTCACGAA |
| *mCol3a1* | Sense | GAGGAATGGGTGGCTATCCG |
| | Antisense | TCGTCCAGGTCTTCCTGACT |
| *mActa2* | Sense | CGAGCGTGAGATTGTCCGT |
| | Antisense | CCCCTGACAGGACGTTGTT |
| *mCtgf* | Sense | AGAACTGTGTACGGAGCGTG |
| | Antisense | GTGCACCATCTTTGGCAGTG |

Uncropped blots

Figure 2c

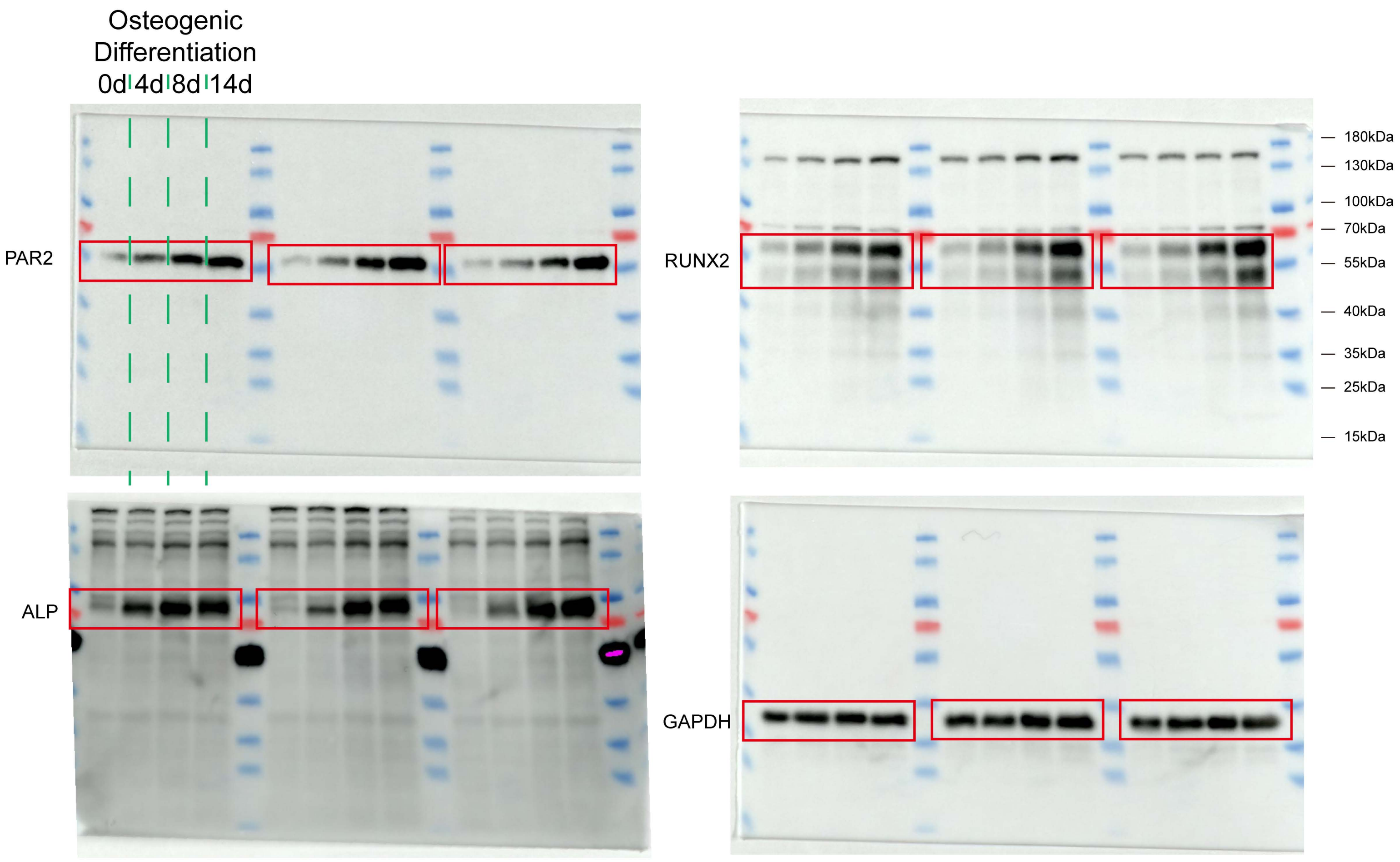

Figure 2d

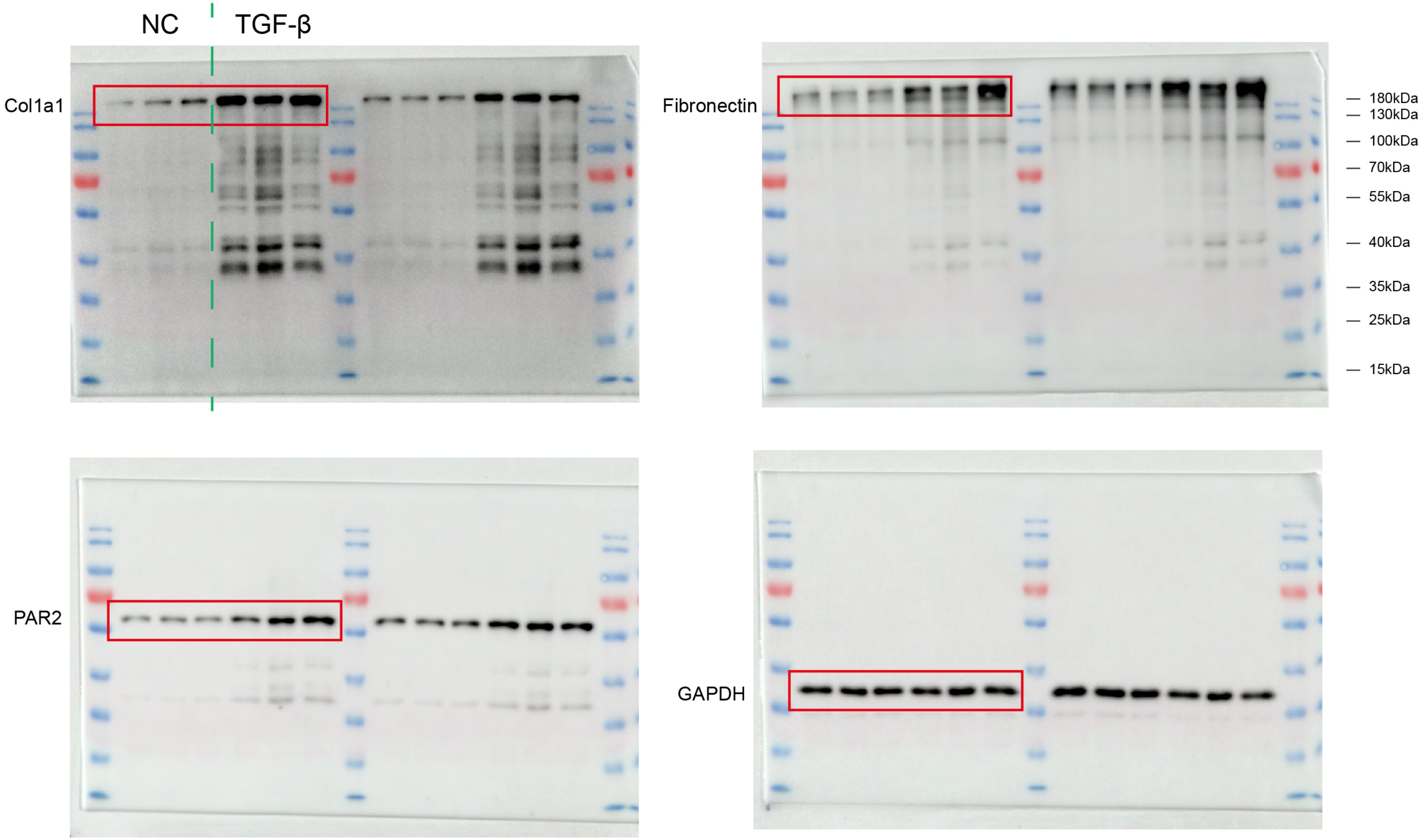

NC
TGF-β
Col1a1
Fibronectin
180kDa
130kDa
100kDa
70kDa
55kDa
40kDa
35kDa
25kDa
15kDa
PAR2
GAPDH

Figure 2f

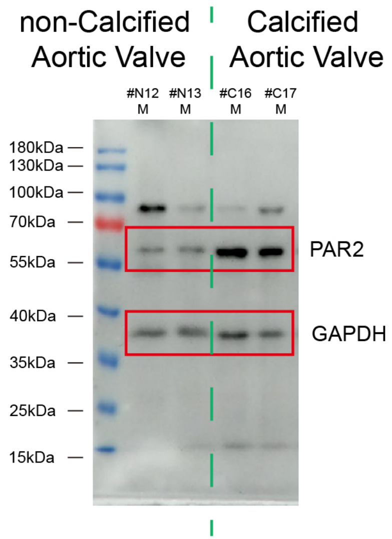

non-Calcified Aortic Valve
Calcified Aortic Valve
#N12 M
#N13 M
#C16 M
#C17 M
180kDa
130kDa
100kDa
70kDa
55kDa
40kDa
35kDa
25kDa
15kDa
PAR2
GAPDH


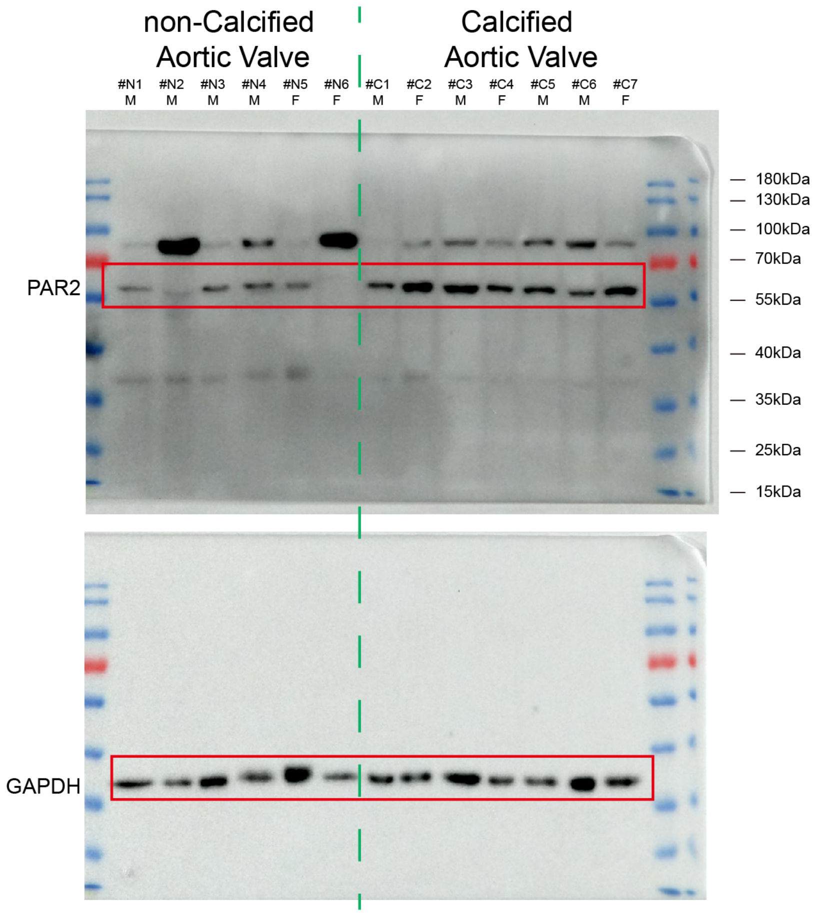

non-Calcified Aortic Valve
Calcified Aortic Valve
#N1 M
#N2 M
#N3 M
#N4 M
#N5 F
#N6 F
#C1 M
#C2 F
#C3 M
#C4 F
#C5 M
#C6 M
#C7 F
PAR2
GAPDH
180kDa
130kDa
100kDa
70kDa
55kDa
40kDa
35kDa
25kDa
15kDa


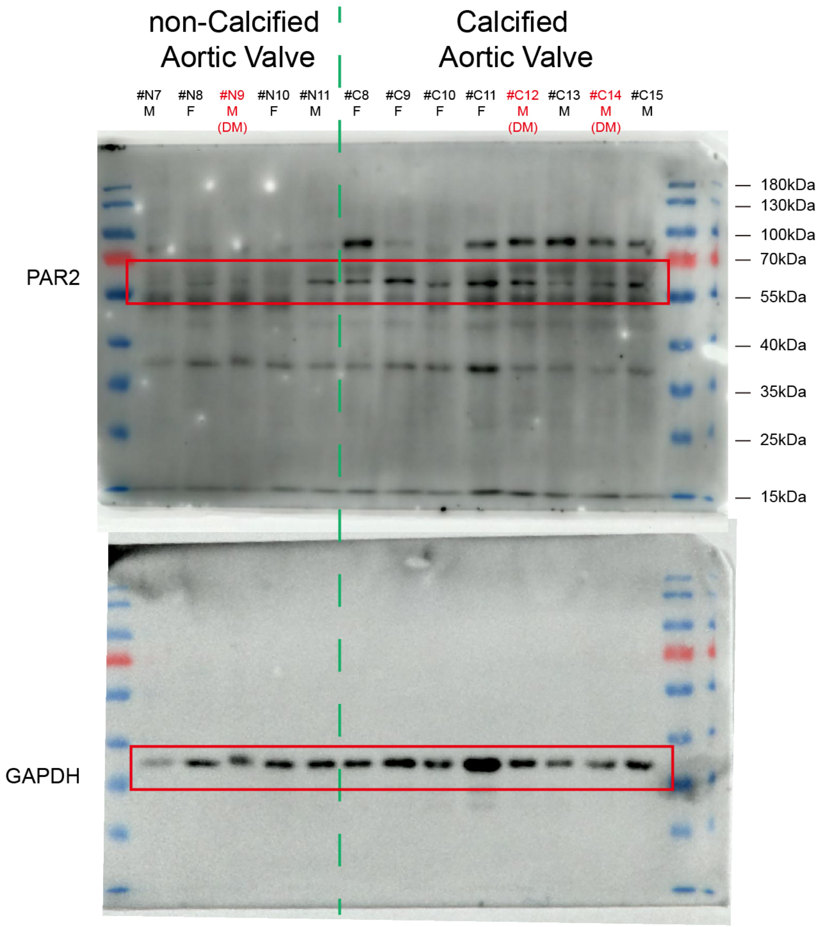

non-Calcified Aortic Valve
Calcified Aortic Valve
#N7 M
#N8 F
#N9 M (DM)
#N10 F
#N11 M
#C8 F
#C9 F
#C10 F
#C11 F
#C12 M (DM)
#C13 M
#C14 M (DM)
#C15 M
PAR2
GAPDH
180kDa
130kDa
100kDa
70kDa
55kDa
40kDa
35kDa
25kDa
15kDa

Figure 2k

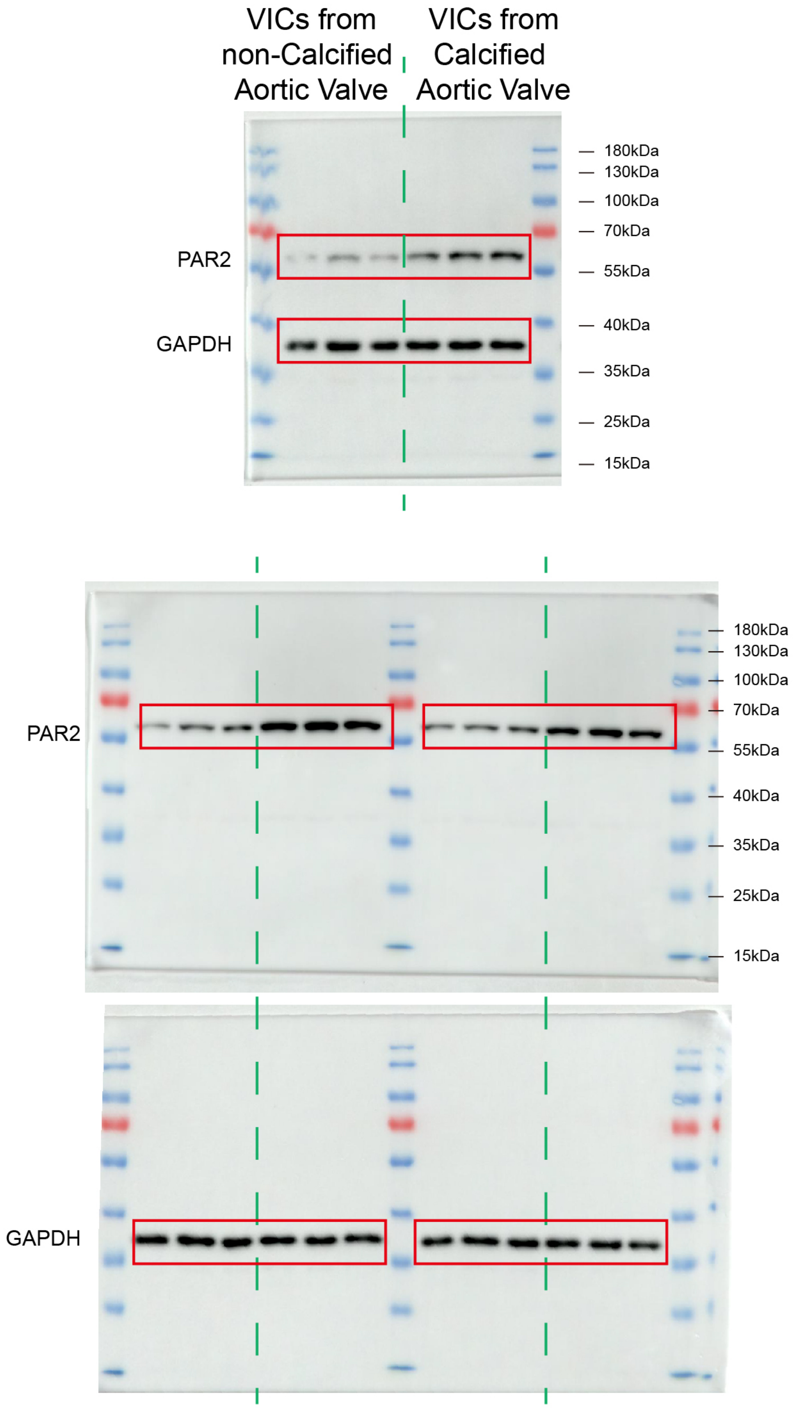
VICs from non-Calcified Aortic Valve
VICs from Calcified Aortic Valve
PAR2
GAPDH
180kDa
130kDa
100kDa
70kDa
55kDa
40kDa
35kDa
25kDa
15kDa
PAR2
180kDa
130kDa
100kDa
70kDa
55kDa
40kDa
35kDa
25kDa
15kDa
GAPDH

Figure 5d

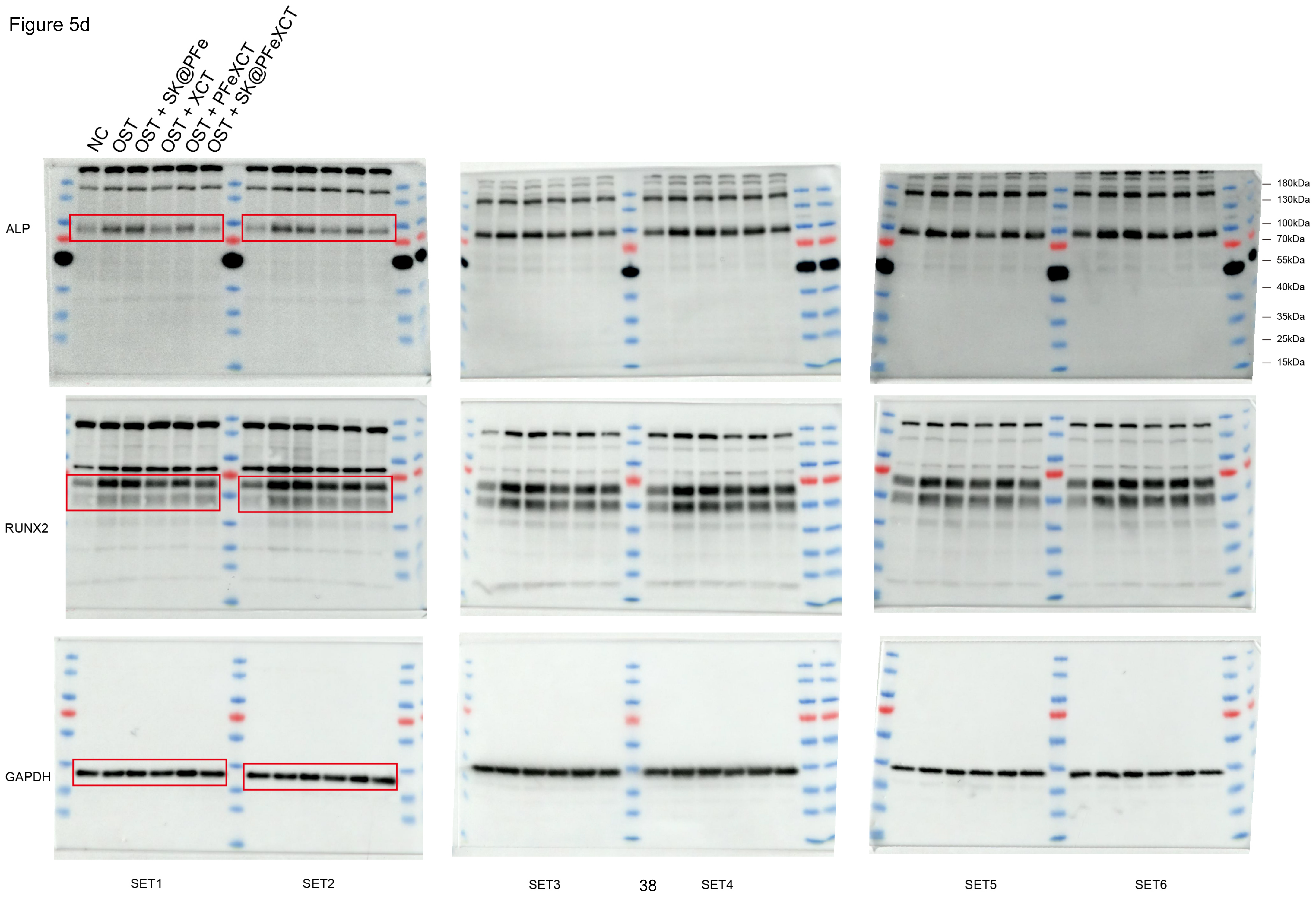
NC
OST
OST + SK@PFe
OST + XCT
OST + PFeXCT
OST + SK@PFeXCT
ALP
RUNX2
GAPDH
180kDa
130kDa
100kDa
70kDa
55kDa
40kDa
35kDa
25kDa
15kDa
SET1
SET2
SET3
SET4
SET5
SET6

Figure 8i

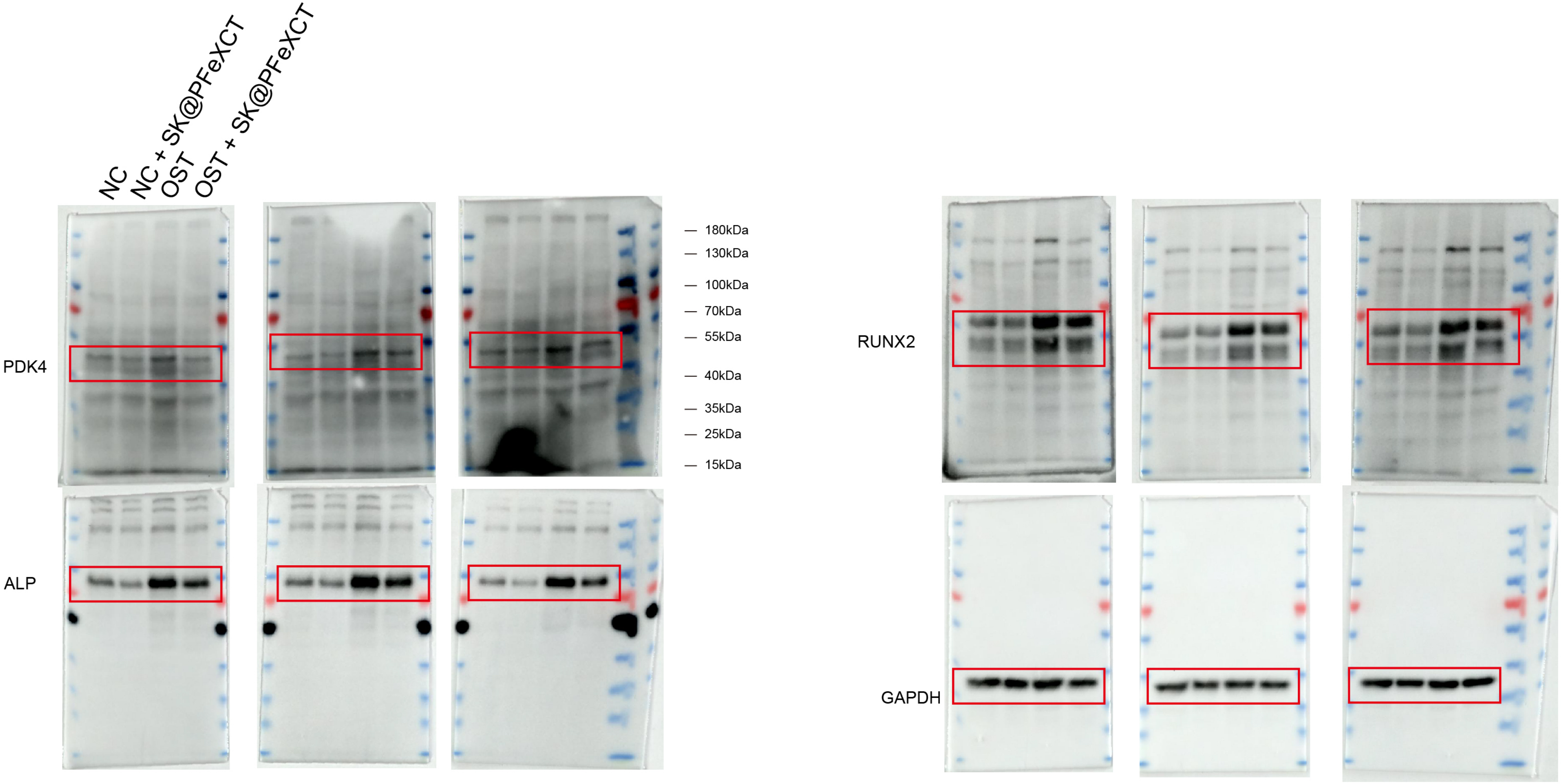
NC
NC + SK@PFeXCT
OST
OST + SK@PFeXCT
PDK4
ALP
180kDa
130kDa
100kDa
70kDa
55kDa
40kDa
35kDa
25kDa
15kDa
RUNX2
GAPDH

Supplementary Fig. 3a

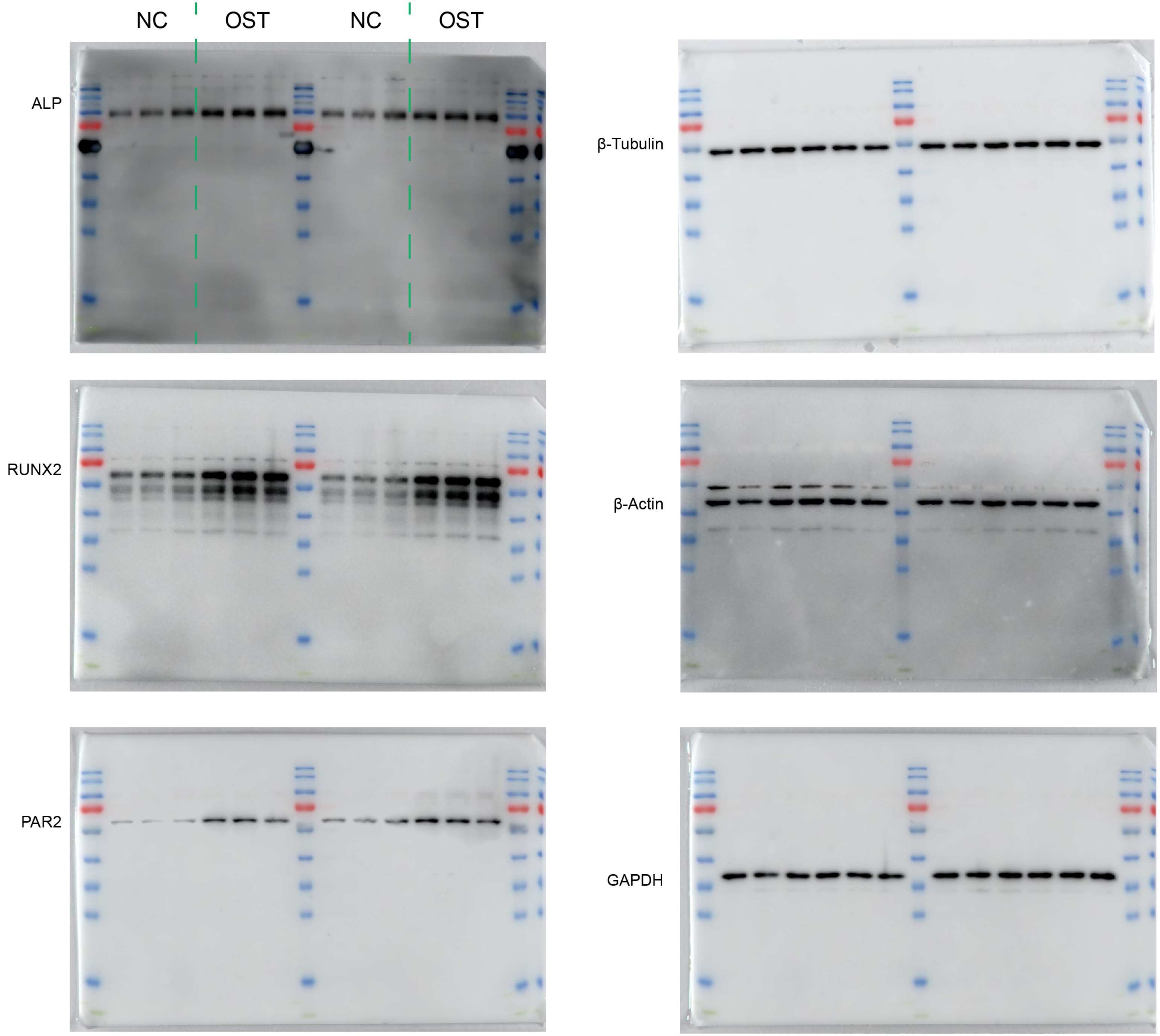

Supplementary Fig. 3b

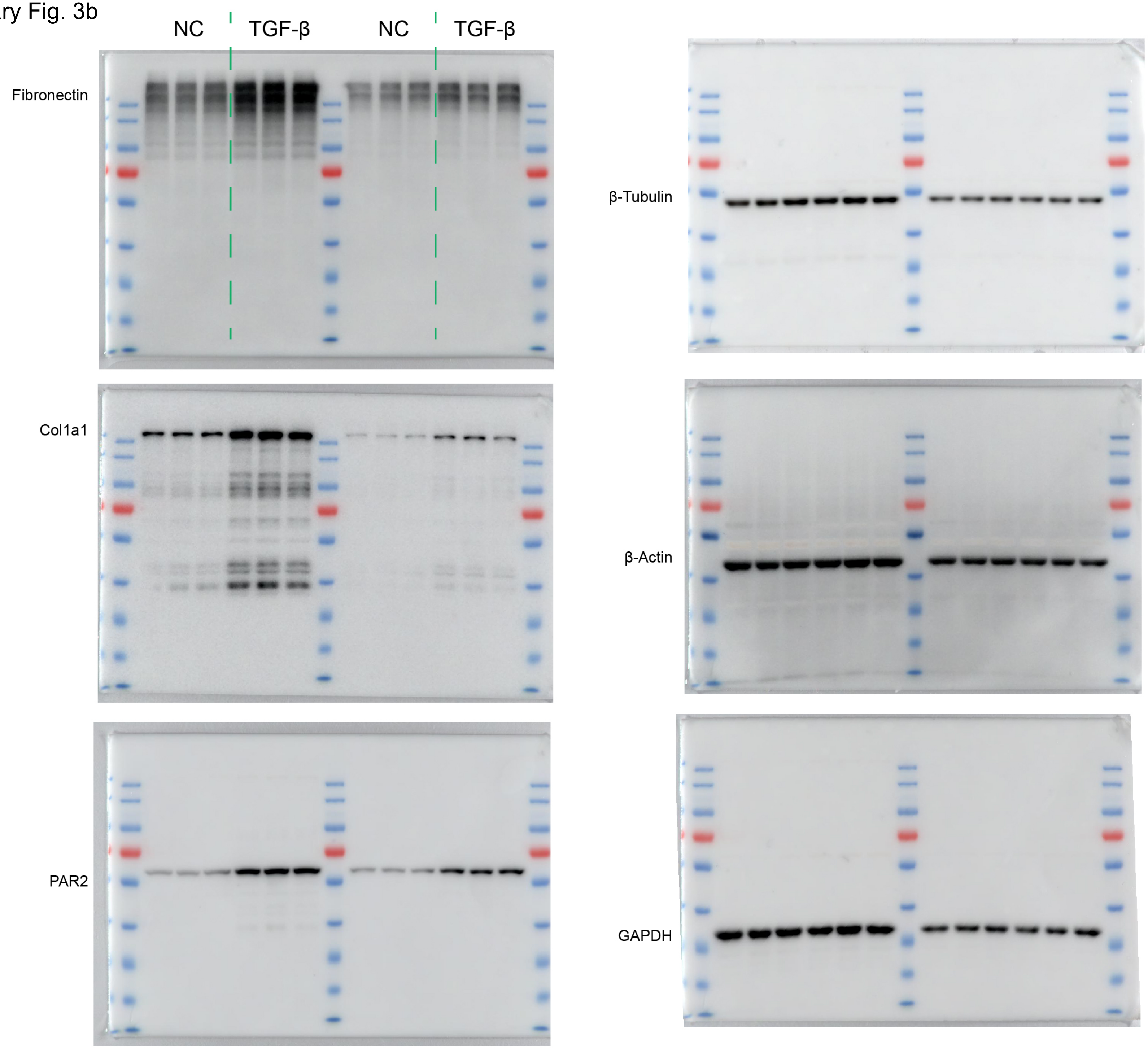

Supplementary Fig. 20c

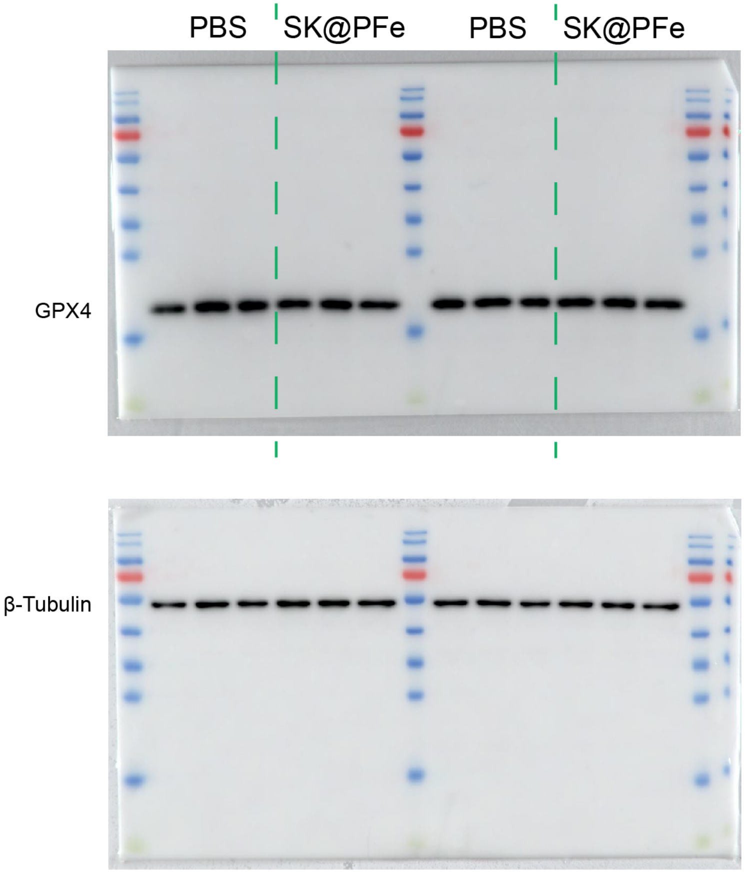


Supplementary Fig. 22

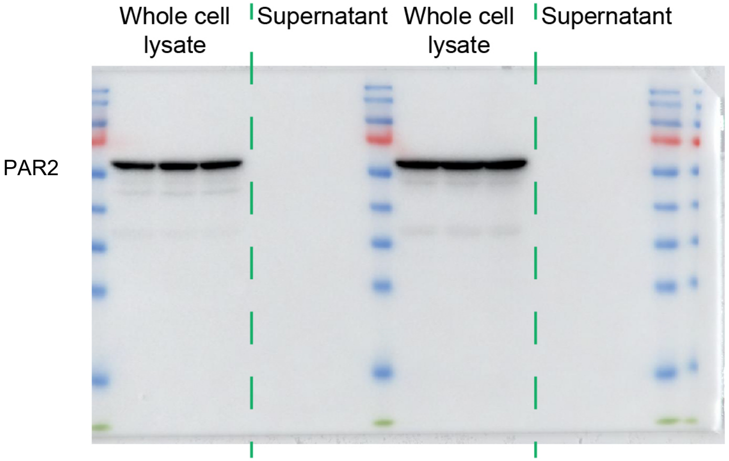

Supplement: Supplementary file 1 — Supplementary information [file 41467_2024_44726_MOESM1_ESM.pdf]
