# Supplementary material for: Enhancing aortic valve drug delivery with PAR2-targeting magnetic nano-cargoes for calcification alleviation
Source: Nat Commun. 2024 Jan 16;15:557. doi: 10.1038/s41467-024-44726-0 (PMC10792006; doi:10.1038/s41467-024-44726-0)
Supplement: Supplementary file 3 — Reporting Summary [file 41467_2024_44726_MOESM3_ESM.pdf]

nature portfolio

Corresponding author(s): Xianbao Liu; Jian`an Wang; Tanchen Ren

Last updated by author(s): Dec 5, 2023

Reporting Summary

Nature Portfolio wishes to improve the reproducibility of the work that we publish. This form provides structure for consistency and transparency in reporting. For further information on Nature Portfolio policies, see our Editorial Policies and the Editorial Policy Checklist.

Statistics

For all statistical analyses, confirm that the following items are present in the figure legend, table legend, main text, or Methods section.

n/a	Confirmed	
☐	☒	The exact sample size (n) for each experimental group/condition, given as a discrete number and unit of measurement
☐	☒	A statement on whether measurements were taken from distinct samples or whether the same sample was measured repeatedly
☐	☒	The statistical test(s) used AND whether they are one- or two-sided<br>*Only common tests should be described solely by name; describe more complex techniques in the Methods section.*
☒	☐	A description of all covariates tested
☐	☒	A description of any assumptions or corrections, such as tests of normality and adjustment for multiple comparisons
☐	☒	A full description of the statistical parameters including central tendency (e.g. means) or other basic estimates (e.g. regression coefficient) AND variation (e.g. standard deviation) or associated estimates of uncertainty (e.g. confidence intervals)
☐	☒	For null hypothesis testing, the test statistic (e.g. F, t, r) with confidence intervals, effect sizes, degrees of freedom and P value noted<br>*Give P values as exact values whenever suitable.*
☒	☐	For Bayesian analysis, information on the choice of priors and Markov chain Monte Carlo settings
☒	☐	For hierarchical and complex designs, identification of the appropriate level for tests and full reporting of outcomes
☒	☐	Estimates of effect sizes (e.g. Cohen's d, Pearson's r), indicating how they were calculated

Our web collection on statistics for biologists contains articles on many of the points above.

Software and code

Policy information about availability of computer code

Data collection	No specialized software was used for data acquisition. LightCycler 480 (Version 1.5) for qPCR, Leica LAS X (Version 3.0) for image acquisition, Living Image (Version 4.4) for ex vivo imaging, ImageQuant 800 for immunoblotting, Visual Sonic Vevo 2100 for Echocardiography, and BD FACSDiva (version 9.0) for flow cytometry.
Data analysis	ImageQuant TL (Version 8.2), Image-Pro Plus (Version 6.0), Leica LAS X (Version 3.0), FlowJo VX (Version 10.0), Seahore Wave Desktop (Version 2.6) and GraphPad (Version 8.1) were used to analyze these data.

For manuscripts utilizing custom algorithms or software that are central to the research but not yet described in published literature, software must be made available to editors and reviewers. We strongly encourage code deposition in a community repository (e.g. GitHub). See the Nature Portfolio guidelines for submitting code & software for further information.

Data

Policy information about availability of data

All manuscripts must include a data availability statement. This statement should provide the following information, where applicable:

- Accession codes, unique identifiers, or web links for publicly available datasets
- A description of any restrictions on data availability
- For clinical datasets or third party data, please ensure that the statement adheres to our policy

The authors confirm that the data supporting the findings of this study are available within the article and its supplementary materials or from the corresponding author upon reasonable request. The data of bulk RNA-seq reported in this paper have been deposited in the GEO under the accession number GSE252268 (https://

www.ncbi.nlm.nih.gov/geo/query/acc.cgi?acc=GSE252268). The publicly available scRNA-seq data used in this study are available in the BioProject database under accession code "PRJNA562645 [https://www.ncbi.nlm.nih.gov/bioproject/?term=PRJNA562645]". Source data are provided with this paper.

Research involving human participants, their data, or biological material

Policy information about studies with human participants or human data. See also policy information about sex, gender (identity/presentation), and sexual orientation and race, ethnicity and racism.

Reporting on sex and gender	The human research participant is not a sex/gender related research and we collected the aortic valve specimens were obtained from 21 male and 11 female patients. All specimens were collected after the patient or their relative signed a written informed consent. Patient information is listed in Supplementary Table 1.
Reporting on race, ethnicity, or other socially relevant groupings	Race, ethnicity, or other socially relevant groupings were not considered in the study design, but due to hospital limitations, the specimens were all collected from Chinese.
Population characteristics	All patient informations are listed in Supplementary Table 1.
Recruitment	All aortic valve specimens in this study were collected unbiasedly from patients undergoing surgery in the Department of Cardiac Surgery, Second Affiliated Hospital of Zhejiang University. Calcified aortic valve leaflets were obtained from severe aortic stenosis patients who underwent aortic valve replacement surgery, while non-calcified aortic valves were obtained from heart transplant recipients with Stanford type A acute aortic dissection or aortic valve regurgitation. Valves from patients with rheumatic disease, infective endocarditis, congenital valvular disease, or diabetes were excluded
Ethics oversight	All experiments involving humans were conducted in accordance with the Declaration of Helsinki and were approved by the Human Research Ethics Committee of the Second Affiliated Hospital, Zhejiang University (No. IRB-2022-0085). Written informed consent was provided by each participant.

Note that full information on the approval of the study protocol must also be provided in the manuscript.

Field-specific reporting

Please select the one below that is the best fit for your research. If you are not sure, read the appropriate sections before making your selection.

☒ Life sciences ☐ Behavioural & social sciences ☐ Ecological, evolutionary & environmental sciences

For a reference copy of the document with all sections, see nature.com/documents/nr-reporting-summary-flat.pdf

Life sciences study design

All studies must disclose on these points even when the disclosure is negative.

Sample size	We did not use statistical method to predetermine sample size. The sample size was based on the previous literature (DOI: 10.1093/eurheartj/ehac818) that a minimum of N = 3 biological replicates with sufficient reproducibility in cell experiments and a minimum of N = 5 biological replicates with sufficient reproducibility in animal experiments. The exact n values are provided in the corresponding figure legends.
Data exclusions	No data were excluded in this study.
Replication	All in vivo and in vitro experiments were indpendently performed at least 3 times. All attempts at replication were successful.
Randomization	In all experiments used in this study, the samples were divided randomly into each different group.
Blinding	Blinding was applicable in this study.

Reporting for specific materials, systems and methods

We require information from authors about some types of materials, experimental systems and methods used in many studies. Here, indicate whether each material, system or method listed is relevant to your study. If you are not sure if a list item applies to your research, read the appropriate section before selecting a response.

Materials & experimental systems

n/a	Involved in the study
☐	☒ Antibodies
☐	☒ Eukaryotic cell lines
☒	☐ Palaeontology and archaeology
☐	☒ Animals and other organisms
☒	☐ Clinical data
☒	☐ Dual use research of concern
☒	☐ Plants

Methods

n/a	Involved in the study
☒	☐ ChIP-seq
☐	☒ Flow cytometry
☒	☐ MRI-based neuroimaging

Antibodies

Antibodies used

- primary antibodies [format: host anti-protein (company, catalog number, dilution, lot number & Clone number if available)]
1. rabbit monoclonal anti-RUNX2 (Huabio, ET1612-47, 1:1000 for Westen-blot and 1:100 for Immunofluorescence, clone SD208-0)
2. mouse monoclonal anti-ALP (R&D, MAB1448, 1:1000 for Westen-blot, clone # B4-78)
3. rabbit monoclonal anti-PAR2 (Abcam, ab180953, 1:1000 for Westen-blot, clone EPR13675)
4. mouse monoclonal anti-PAR2 (Invitrogen, 35-2300, 1:200 for Immunofluorescence, 1:200 for immunohistochemistry , clone SAM11)
5. rabbit polyclonal anti-PDK4 (Proteintech, 12949-1-AP, 1:1000 for Westen-blot and 1:100 for Immunofluorescence)
6. rabbit polyclonal anti-COL1A1 (Invitrogen, PA5-86949, 1:1000 for Westen-blot)
7. rabbit monoclonal anti-Fibronectin (Abcam, ab268020, 1:1000 for Westen-blot, clone EPR23110-46)
8. rabbit monoclonal anti-GPX4 (Abcam, ab125066, 1:1000 for Westen-blot, clone EPNCIR144)
9. HRP-Conjugated rabbit monoclonal anti-GAPDH (Huabio, ET1702-66, 1:5000 for Westen-blot, clone JF81-04)
10.HRP-Conjugated rabbit monoclonal anti-beta Actin (Huabio, ET1702-67, 1:5000 for Westen-blot, clone JF53-10)
11.HRP-Conjugated rabbit monoclonal anti-beta Tubulin (Huabio, ET1702-68, 1:5000 for Westen-blot, clone JF41-50)
12. mouse monoclonal anti-alpha smooth muscle Actin (Abcam, ab7817, 1:200 for Immunofluorescence, clone 1A4)
13. rabbit monoclonal anti-VE-Cadherin (CST, 2500T, 1:400 for Immunofluorescence, clone D87F2)
14. goat polyclonal anti-Vimentin (Abcam, ab11256, 1:200 for Immunofluorescence)
15.rabbit polyclonal anti-CD31((Proteintech,11265-1-AP, 1:200for Immunofluorescence)
16. rabbit monoclonal anti-Osterix (Abcam, ab209484, 1:300 for Immunofluorescence, clone EPR21034)
17. rabbit monoclonal anti-Bmp2 (Abcam, ab284387, 1:100 for Immunofluorescence, clone EPR24209-61)

- secondary antibodies [format: host anti-protein (company, catalog number, dilution, lot number if available)]:
18. goat anti-rabbit HRP conjugated (CST, 7074S, 1:3,000).
19. horse anti-mouse HRP conjugated (CST, 7076S, 1:3,000).
20. goat anti-rabbit Alexa 488 conjugated (Invitrogen, A-11008, 1:300)
21. goat anti-mouse Alexa 488 conjugated (Invitrogen, A-11001, 1:300)
22. donkey anti-mouse Alexa 488 conjugated (Invitrogen, A-21202, 1:300)
23. donkey anti-rabbit Alexa 555 conjugated (Invitrogen, A-31572, 1:300)
24. donkey anti-goat Alexa 647 conjugated (Invitrogen, A-21447, 1:300)

Validation

All the antibodies used in this study are commercially available, validated by the manufactures or by our laboratory:
1. rabbit monoclonal anti-RUNX2 (http://www.huabio.cn/product/RUNX2-antibody-ET1612-47)
2. mouse monoclonal anti-ALP (https://www.rndsystems.com/products/human-alkaline-phosphatase-alpl-antibody-b4-78_mab1448)
3. rabbit monoclonal anti-PAR2 (https://www.abcam.com/products/primary-antibodies/par2-antibody-epr13675-ab180953.html)
4. mouse monoclonal anti-PAR2 (https://www.thermofisher.cn/cn/zh/antibody/product/PAR2-Antibody-clone-SAM11-Monoclonal/35-2300)
5. rabbit polyclonal anti-PDK4 (https://www.ptgcn.com/products/PDK4-Antibody-12949-1-AP.htm)
6. rabbit polyclonal anti-COL1A1 (https://www.thermofisher.cn/cn/zh/antibody/product/COL1A1-Antibody-Polyclonal/PA5-86949)
7. rabbit monoclonal anti-Fibronectin (https://www.abcam.cn/products/primary-antibodies/fibronectin-antibody-epr23110-46-ab268020.html)
8. rabbit monoclonal anti-GPX4(https://www.abcam.cn/products/primary-antibodies/glutathione-peroxidase-4-antibody-epncir144-ab125066.html)
9. HRP-Conjugated rabbit monoclonal anti-GAPDH (https://www.huabio.com/products/gapdh-hrp-conjugated-antibody-clone-jf81-04-recombinant-monoclonal-et1702-66)
10.HRP-Conjugated rabbit monoclonal anti-beta Actin (http://www.huabio.cn/product/beta-Actin-HRP-conjugated-antibody-ET1702-67)
11.HRP-Conjugated rabbit monoclonal anti-beta Tubulin (http://www.huabio.cn/product/beta-Tubulin-HRP-conjugated-antibody-ET1702-68)
12. mouse monoclonal anti-alpha smooth muscle Actin (https://www.abcam.cn/products/primary-antibodies/alpha-smooth-muscle-actin-antibody-1a4-ab7817.html)
13. rabbit monoclonal anti-VE-Cadherin (https://www.cellsignal.com/products/primary-antibodies/ve-cadherin-d87f2-xp-rabbit-mab/2500)
14. goat polyclonal anti-Vimentin (https://www.abcam.cn/products/primary-antibodies/vimentin-antibody-ab11256.html)
15. rabbit polyclonal anti-CD31 (https://www.ptgcn.com/products/PECAM1-Antibody-11265-1-AP.htm)

16. rabbit monoclonal anti-Osterix (https://www.abcam.cn/products/primary-antibodies/sp7--osterix-antibody-epr21034-ab209484.html)
17. rabbit monoclonal anti-Bmp2 (https://www.abcam.cn/products/primary-antibodies/bmp2-antibody-epr24209-61-ab284387.html)
18. goat anti-rabbit HRP conjugated (https://www.cellsignal.cn/products/secondary-antibodies/anti-rabbit-igg-hrp-linked-antibody/7074).
19. horse anti-mouse HRP conjugated (https://www.cellsignal.cn/products/secondary-antibodies/anti-rabbit-igg-hrp-linked-antibody/7076).
20. goat anti-rabbit Alexa 488 conjugated (https://www.thermofisher.cn/cn/zh/antibody/product/Goat-anti-Rabbit-IgG-H-L-Cross-Adsorbed-Secondary-Antibody-Polyclonal/A-11008)
21. goat anti-mouse Alexa 488 conjugated (https://www.thermofisher.cn/cn/zh/antibody/product/Goat-anti-Mouse-IgG-H-L-Cross-Adsorbed-Secondary-Antibody-Polyclonal/A-11001)
22. donkey anti-mouse Alexa 488 conjugated (https://www.thermofisher.cn/cn/zh/antibody/product/Donkey-anti-Mouse-IgG-H-L-Highly-Cross-Adsorbed-Secondary-Antibody-Polyclonal/A-21202)
33. donkey anti-rabbit Alexa 555 conjugated (https://www.thermofisher.cn/cn/zh/antibody/product/Donkey-anti-Rabbit-IgG-H-L-Highly-Cross-Adsorbed-Secondary-Antibody-Polyclonal/A-31572)
24. donkey anti-goat Alexa 647 conjugated (https://www.thermofisher.cn/cn/zh/antibody/product/Donkey-anti-Goat-IgG-H-L-Cross-Adsorbed-Secondary-Antibody-Polyclonal/A-21447)

Eukaryotic cell lines

Policy information about cell lines and Sex and Gender in Research

Cell line source(s)	The mouse aortic vascular smooth muscle cell line MOVAS-1 was purchased from Sunncell, Wuhan,China.
Authentication	no authentication has been used.
Mycoplasma contamination	Mycoplasma contamination test is negative.
Commonly misidentified lines (See ICLAC register)	No commonly misidentified lines were used in this study.

Animals and other research organisms

Policy information about studies involving animals; ARRIVE guidelines recommended for reporting animal research, and Sex and Gender in Research

Laboratory animals	Eight-week-old wild-type C57BL/6J mice were purchased from Vital River Laboratories (Beijing, China), and eight-week-old low-density lipoprotein receptor (Ldlr)-/- mice in the C57BL/6J background were purchased from GemPharmatech (Nanjing, China). Mice were housed in a specific pathogen-free environment with a standard 12-h light/12-h dark conditions and had free access to water and food. The room temperature was maintained at 20~26°C, and relative humidity was around 40%~70%.
Wild animals	No wild animals were used in the study.
Reporting on sex	Based on previous animal studies of aortic valve calcification (https://doi.org/10.1038/s41467-022-33202-2,doi: 10.1093/EURHEARTJ/EHAC818.) we selected male mice for the construction of CAVD models. Higher estrogen level in female mice affects CAVD progression in mice and may complicate the date interpretation.
Field-collected samples	No field collected samples were used in the study.
Ethics oversight	All animal experiments were performed in accordance with the Guide for the Care and Use of Laboratory Animals published by the NIH and approved by the Animal Use Committee of The Second Affiliated Hospital, Zhejiang University (No. AIRB-2022-0109).

Note that full information on the approval of the study protocol must also be provided in the manuscript.

Flow Cytometry

Plots

Confirm that:

- [x] The axis labels state the marker and fluorochrome used (e.g. CD4-FITC).
- [x] The axis scales are clearly visible. Include numbers along axes only for bottom left plot of group (a 'group' is an analysis of identical markers).
- [x] All plots are contour plots with outliers or pseudocolor plots.
- [x] A numerical value for number of cells or percentage (with statistics) is provided.

Methodology

Sample preparation	hVICs were seeded in six well plate and cultured with SK@PFeCy5 or PFeCy5 nanoparticles for 6 hours, then digested with trypsin and washed with HBSS. Single-cell fluorescence intensity was measured using flow cytometry (LSRFortessa, BD, USA).
Instrument	BD LSRFortessa
Software	BD FACSDiva (version 9.0) and FlowJo VX (Version 10.0)
Cell population abundance	Cells were not grouped in this study.
Gating strategy	After excluding dead cell debris and adhesion cells, and all single viable cells were used to count the mean fluorescence intensity.

☒ Tick this box to confirm that a figure exemplifying the gating strategy is provided in the Supplementary Information.
